# Characterization of Monogenic Kidney Disease in Older Patients With CKD

**DOI:** 10.1016/j.ekir.2025.04.017

**Published:** 2025-04-22

**Authors:** Elhussein A.E. Elhassan, Sarah Cormican, Shohdan M. Osman, Sahin Sarihan, Omri Teltsh, Fergus E. Poynton, Matthew D. Griffin, Liam Casserly, Emma McCann, Anthony J. Bleyer, Stanislav Kmoch, Martina Živná, Katherine A. Benson, Gianpiero L. Cavalleri, Peter J. Conlon

**Affiliations:** 1 Department of Nephrology and Transplantation, Beaumont Hospital, Dublin, Ireland; 2 Department of Medicine, Royal College of Surgeons in Ireland, Dublin, Ireland; 3 School of Pharmacy and Biomolecular Sciences, Royal College of Surgeons, Dublin, Ireland; 4 Regenerative Medicine Institute at CÚRAM Research Center for Medical Devices, School of Medicine, University of Galway, Ireland; 5 Nephrology Department, Galway University Hospitals, Saolta University Healthcare Group, Galway, Ireland; 6 Department of Renal Medicine, University Hospital Limerick, Limerick, Ireland; 7 The Department of Clinical Genetics, Children's Health Ireland at Crumlin, Dublin, Ireland; 8 Research Unit for Rare Diseases, Department of Pediatrics and Inherited Metabolic Disorders, First Faculty of Medicine, Charles University in Prague, Czech Republic; 9 Section on Nephrology, Wake Forest School of Medicine, Winston-Salem, North Carolina; 10 FutureNeuro SFI Research Center, Royal College of Surgeons, Dublin, Ireland

**Keywords:** CKD, monogenic, MPS, older, polycystic kidney disease

## Abstract

**Introduction:**

Although 10% of adults with chronic kidney disease (CKD) have a monogenic cause, the characteristics of monogenic CKD in older adults (aged ≥ 60 years) are less characterized. We aimed to assess the clinical and genetic spectrum of older adults with CKD and the clinical utility of genetic findings.

**Methods:**

The diagnostic yield of clinically validated disease-causing variants and their type (“typical” vs. “later-onset” phenotypes) were analyzed in older patients with suspected monogenic CKD who were referred to an Irish registry according to predetermined criteria. Independent genetic diagnosis and kidney survival time predictors were analyzed using marginal logistic and Cox regression analyses.

**Results:**

Two hundred sixty-five adults (from 202 families) were aged ≥ 60 years at the time of genetic testing, of which 74.3% (197/265) progressed to kidney failure. Diagnostic variants were found in 60.4% (122/202) families, including 39% of noncystic kidney disease families. Variants causing “later-onset” phenotypes were more prevalent in patients with disease-onset ≥ 60 years (56% vs. 8.3%; *P ≤* 0.001), which include genetic variants in: *IFT140*, *ALG5*, *ALG9*, *DNAJB11*, *COL4A5* in females, monoallelic *COL4A3*, and the *UMOD* p.Thr62Pro variant, associated with delayed onset of kidney failure compared with “typical” variants (hazard ratio: 0.52; 95% confidence interval: 0.27–0.98; *P =* 0.043). A family history of CKD and *a priori* cystic kidney disease diagnosis independently predicted genetic diagnosis (*P ≤* 0.05). In 24% of older adults with positive results, the treatment plan was modified.

**Conclusion:**

In older patients with CKD, genetic testing revealed enriched variants associated with less-penetrant phenotypes, often with a family history of CKD, which affects clinical management.

## Graphical abstract

**Figure:**
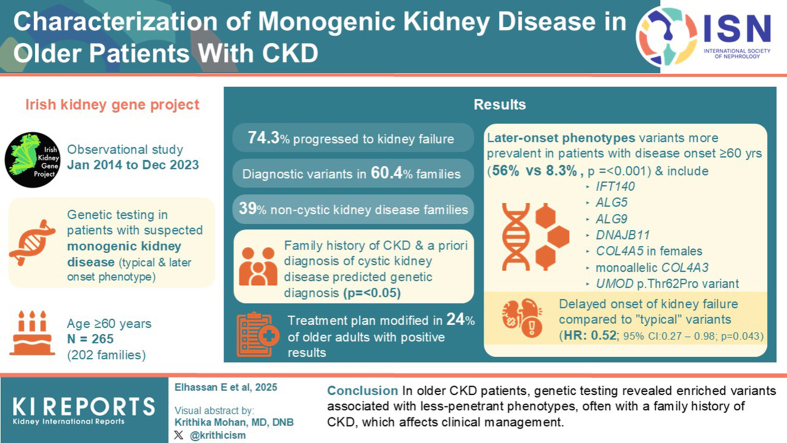

CKD has a global prevalence of approximately 10%,^1^, ^2^, ^3^ and represents a major cause of morbidity and mortality, which imposes a significant economic cost to health care systems.^4^^,^^5^ The etiology of familial clusters of CKD and kidney failure (i.e., death from kidney disease or requiring dialysis or transplantation) can be elucidated through genomic testing, which can determine the relative contributions of monogenic burden.^6^^,^^7^ With the emergence of massive parallel sequencing techniques, over 600 genes have been implicated in genetic kidney disease (monogenic), representing a significant cause of progressive glomerular filtration rate decline and kidney failure.^7^, ^8^, ^9^ It is estimated that genetic kidney disease affects 50% to 70% of children with CKD and 10% to 15% of adults, with congenital anomalies of the kidney and urinary tract, and autosomal dominant polycystic kidney disease (ADPKD) being the most frequently diagnosed disorders,.^7^^,^^10^^,^^11^

Population growth has contributed to an increase in the incidence of CKD, including among older adults.^12^ Genetic kidney disease may also be a common but undiagnosed cause of CKD in older patients.^13^ High cost, access to technology and clinical genetics experts, and lack of genetic knowledge in test selection and interpretation prevent genetic testing from being routinely used in clinical practice.^14^, ^15^, ^16^ This can lead to a bias toward younger adults in referrals for genetic testing.^17^ For example, ADPKD and autosomal dominant tubulointerstitial kidney disease may only present in middle age.^18^, ^19^, ^20^ Moreover, identifying a monogenic cause of CKD offers numerous advantages.^14^^,^^16^ It enables family members of reproductive age to seek information about their reproductive choices. Although family planning may be less relevant to older adults, genetic results may inform the index patient about prognosis, enhance clinical understanding of disease pathophysiology, and lead to new targeted therapies.^21^

Although age-related decline in estimated glomerular filtration rate partially explains CKD in older adults, longer lifespans allow pathogenic variants with a later disease-onset and less-penetrant phenotypes to result in pathologic changes and loss of kidney function in older adults with CKD, and have consequences on the whole family.^22^, ^23^, ^24^ Several studies have examined glomerulonephritis (GN),^25^ CKD,^26^^,^^27^ and renal histopathology^28^ in older patients; however, the range of genetic kidney disease in older patients remains largely underexplored.^29^

Here, we present the genetic characterization and clinical outcomes of adults aged ≥ 60 years who underwent genetic testing for a wide range of kidney diseases as part of the Irish Kidney Gene Project.

## Methods

### Study Design and Cohort Characterization

Clinical and sequencing data from the Irish Kidney Gene Project, a national research study established to investigate monogenic kidney diseases, were utilized. In this observational cohort study, information on all recruited individuals across 24 participating nephrology clinics and dialysis units from January 19, 2014 to December 31, 2023, was evaluated. Referred individuals were assessed for eligibility using predetermined inclusion and exclusion criteria as outlined in a written protocol. The inclusion criteria were as follows: (i) adult participants (aged ≥ 18 years) and 1 or more of the following: (ii) a clinically suspected monogenic genetic kidney disease with or without extrarenal phenotypic features (such as hearing loss and eye or retinal involvement [see *a priori* clinical information below]), (iii) a family history of kidney disease, (iv) reaching kidney failure under the age of 50 years, or (v) no identifiable cause of their CKD. Individuals with an existing kidney clinical or phenotypic diagnosis, such as likely or proven diabetic nephropathy, established biopsy-proven paraproteinemia, nephrotoxin or drug-induced kidney dysfunction, or obstructive uropathy, were excluded from genetic testing. Participants who met the selection criteria for the Irish Kidney Gene Project were genetically tested after obtaining informed consent, which included genetic counseling.

To evaluate the diagnostic yield and spectrum of genetic kidney disease in older patients, participants (probands and their affected relatives) were eligible for the present study if they were aged ≥ 60 years at the time of genetic sequencing. We also included affected participants aged ≥ 60 years who were referred for familial evaluation of younger probands (i.e., tested at age < 60 years). Individuals who were aged < 60 years and those who underwent genetic sequencing at age < 60 years were excluded. Ethical approval was obtained through the Beaumont Hospital Research Ethics Committee (REC_19/28).

According to the *a priori* clinical information of the referring physician and the assessment of available phenotypic data before genetic testing, kidney phenotypes were classified into 6 categories as follows:

1. Cystic kidney disease comprises individuals with multiple cystic kidneys, with or without the presence of a family history, and includes ADPKD.
2. Tubulointerstitial kidney disease comprises individuals with bland urine and a family history of CKD and/or biopsy findings of chronic tubulointerstitial nephritis without a clear precipitating cause.
3. Alport disease and focal segmental glomerulosclerosis (FSGS) included individuals with clinical and/or histological suspicion of Alport disease and those with nephrotic syndrome or nephrotic-range proteinuria and histopathological biopsy findings of FSGS.
4. Chronic GN involves those with membranoproliferative GN, hemolytic uremic syndrome, familial IgA nephropathy, familial GN, and familial amyloid.
5. “Other” comprised individuals with nephrolithiasis, nephrocalcinosis or tubulopathy, and nonsyndromic congenital anomalies of the kidney and urinary tract.
6. Nephropathy of undetermined cause included individuals without a specific or plausible kidney diagnosis (e.g., hypertensive nephropathy, bilateral small kidneys, and nonspecific histopathological diagnosis).

From enrolment, prospectively inputted demographic data (sex at birth, age, ethnicity, age at diagnosis, and age at study recruitment), clinical information, and medical history (including biochemical, imaging, and histological findings, when available, family history of CKD, and progression of kidney disease) were collected and entered into a nationwide nephrology bespoke database (eMEDRenal Version 3.2.4). Self-reported ethnicity was employed to determine the ancestry of individuals at the time of recruitment. If necessary, further information was obtained through scheduled outpatient clinic visits and medical records review. The severity of CKD was graded using the Kidney Disease: Improving Global Outcomes guidelines.^30^ Kidney failure was defined as the commencement of chronic dialysis or preemptive kidney transplantation. Patients were divided into 5 age groups (60 to < 65, 65 to < 70, 70 to < 75, 75 to < 80, and ≥ 80 years).

To assess the diagnostic yield, participants (probands and their affected relatives) were eligible for this study if they had a suspected genetic kidney disease (as defined above) and underwent sequencing at the age of at least ≥ 60 years. The initial patient who underwent genetic evaluation was referred to as the “proband,” whereas “affected relatives” were first- or second-degree relatives aged ≥ 60 years with genetic kidney disease.

### The Clinical Utility of Genetic Results

We evaluated the clinical implications of identifying disease-causing variants in older patients (diagnostic impact). Next, we assessed the clinical impact on the individual's treatment plan. We grouped the treatment plan into the following 5 domains: (i) modifying the treatment plan, which includes assessing extrarenal involvement, avoiding diagnostic kidney biopsy, or avoiding unnecessary medications such as steroids; (ii) assessing disease progression risk; (iii) familial cascade testing; (iv) facilitating kidney transplant evaluation and follow-up, including donor assessment; and (v) referring for genetic counseling (i.e., counseling probands and affected relatives). We also compared the impact of molecular diagnosis on the final pathological diagnosis in patients who have undergone a diagnostic native kidney biopsy before genetic sequencing (pathogenetic impact).

### Genetic Testing–Variant Analysis, Selection, and Interpretation

DNA was extracted from peripheral blood samples using QIA symphony Mini Kit 96 (Qiagen, Germany), or saliva samples using Oragene OG-600 (DNA Genotek, Inc., Ottawa, Ontario, Canada) or GeneFix (Cell Projects, Kent, UK) kits, by standard methods. Genetic analyses were performed using various massive parallel sequencing technologies, including targeted gene panel,^31^ exome sequencing, and *MUC1* genotyping and immunostaining.^32^, ^33^, ^34^ The Genome Analysis Toolkit 4 pipeline was employed for bioinformatic analyses and variant calling.^35^

Genetic variants were classified into 5-tier categories according to the American College of Medical Genetics and Genomics criteria, which were determined by the typical types of variant evidence (e.g., population data, computational data, functional data, and segregation data).^36^ Variants classified as likely pathogenic or pathogenic variants were considered disease-causing.^36^ Variants were reported using the RefSeq Select transcripts, and existing database entries were checked using gnomAD V4.0.0 (http://gnomad.broadinstitute.org) and ClinVar (last accessed on April 29, 2024). Based on phenotypic disease severity, variants were categorized into variants causing a “typical” phenotype (associated with more severe disease with earlier disease-onset) and a “later disease-onset” phenotype (associated with less-penetrant disease). When a genetic diagnosis was made, all disease-causing variants were clinically validated by a Clinical Laboratory Improvement Amendments–approved laboratory and with independent interpretation by a clinical geneticist. Counseling was provided to the patients, and as required, to associated family members.

### Statistical Analysis

The baseline clinical and genetic characteristics were analyzed using descriptive statistics. Based on data distribution, statistics were provided as counts and frequencies for categorical variables and means (±SD) and medians (interquartile range) for continuous variables. Fisher exact test was conducted to evaluate categorical data. The factors associated with establishing a molecular genetic diagnosis were assessed by fitting them with a generalized estimating equation and population average model, which was adjusted for sex and familial cases, because patients from the same family were included. Family identifiers were implemented to account for familial clustering as a random variable. The factors associated with disease progression to kidney failure were investigated using Cox proportional-hazards models. All tests and graphic visualization were performed using STATA SE software version 18.0 (Stata Corp, College Station, TX).

## Results

### Overall Characteristics of Clinically Diagnosed Cohort With CKD

**Figure 1:**
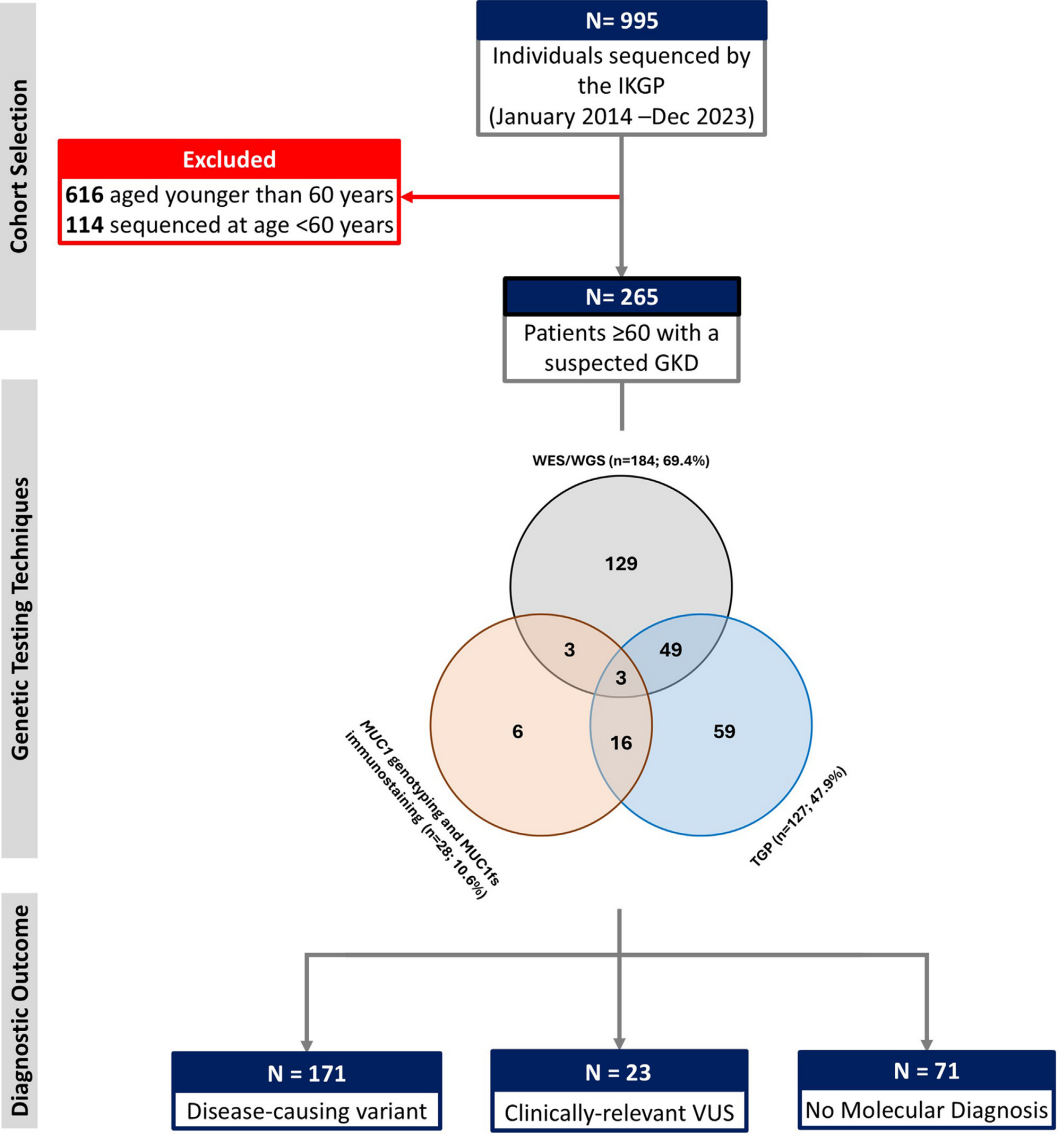
Flowchart of the study. Genetic testing for older adults (aged ≥ 60 years at the time of sequencing) was implemented, taking into account their *a priori* clinical diagnosis and initial diagnostic results (refer to the Venn diagram). GKD, genetic kidney disease; IKGP, the Irish Kidney Gene Project; MPS, massive parallel sequencing; *MUC1*, mucin 1; TGP, targeted gene panel; VUS, variant of uncertain significance; WES, whole exome sequencing; WGS, whole genome sequencing.

The Irish Kidney Gene Project performed genetic sequencing on 995 adult patients suspected of having genetic kidney disease through December 31, 2023. Individuals who underwent sequencing before reaching the age of 60 years (*n* = 730) were excluded, leaving 202 families (265 individuals aged ≥ 60 years) included in this study, with a median age of 71 years (interquartile range: 67–76). Probands and their affected relatives comprised the majority of families (171 [84.7%] families), whereas the remaining families involved older affected relatives who were referred for familial evaluation of probands tested at age < 60 years. All patients self-reported as Caucasian, and 50.6% [134/265] were female. The median age at disease-onset was 44 (interquartile range: 30–57) years, and 55 individuals (20.7%) had a disease-onset at age ≥ 60 years. The flow diagram of the study cohort is illustrated in Figure 1. A family history of kidney disease was reported in 83.7%. The prevalence of hypertension was 89.4%, and diabetes was 12.1%. At the last assessment, about three-quarters of this cohort (74.3%) had progressed to kidney failure at a mean age of 56.4 ± 12.2 years. In Table 1, we show the baseline characteristics of the patients stratified by their genomic outcome status.

**Table 1:** Phenotypic characteristics of the 265 affected individuals aged > 60 years sequenced by the Irish Kidney Gene Project

Phenotypic characteristics	Total	Monogenic Dx	No Genetic Dx
Number of families / individuals, *n*^a^	202 / 265	122 / 171	80 / 94
Age, median (IQR), (yrs)	71 (67–76)	71 (67–76)	72 (68–76)
Sex at birth, *n* (%)			
Female	134 (50.6)	93 (54.4)	41 (43.6)
Male	131 (49.4)	78 (45.6)	53 (56.4)
Age groups, *n* (%), yrs			
60 to < 65	27 (10.2)	20 (11.7)	7 (7.4)
65 to < 70	79 (29.8)	53 (31)	26 (27.7)
70 to < 75	72 (21.2)	45 (26.3)	27 (28.7)
75 to < 80	52 (19.6)	29 (17)	23 (24.5)
≥ 80	35 (13.2)	24 (14)	11 (11.7)
Family history of kidney disease, *n* (%)			
Yes	222 (83.7)	157 (91.8)	65 (69.2)
No	43 (16.3)	14 (8.2)	29 (30.8)
Hypertension, *n* (%)			
Yes	237 (89.4)	162 (94.7)	75 (79.8)
No	10 (3.8)	2 (1.2)	8 (8.5)
Missing data	18 (6.8)	7 (4.1)	11 (11.7)
Diabetes mellitus, *n* (%)			
Yes	32 (12.1)	23 (13.5)	9 (9.6)
No	209 (78.8)	137 (80.1)	72 (76.6)
Missing data	24 (9.1)	11 (6.4)	13 (13.8)
Age at disease onset, median (IQR) (yrs)^b^	44 (30–57)	44 (30–55)	45 (27–60)
Renal status^c^			
Progression to kidney failure, *n* (%)	197 (74.3)	133 (77.8)	64 (68.1)
Age at kidney failure, mean ± SD	56.4 ± 12.2	57 ± 10.9	55.3 ± 14.6
Median age of kidney survival, yrs (95% CI)^d^	62 (53–73)	61 (52–72)	66 (54–81)
CKD^e^	68 (25.7)	38 (22.2)	30 (31.9)
G1–G3a	17 (6.4)	12 (7)	5 (5.3)
G3b–G5	33 (12.5)	18 (10.5)	15 (16)
Missing data	18 (6.8)	8 (4.7)	10 (10.6)
Age at sequencing, median (IQR), yrs	67 (63–72)	67 (63–73)	67 (62–70)
Individuals with kidney failure at genetic testing, *n* (%)	170 (64.2)	114 (66.7)	56 (59.6)
Kidney biopsy (yes), *n* (%)^f^	45 (17)	18 (11.1)	27 (28.7)
Histological categories			
Glomerular disease	16 (40)	2 (22.2)	14 (51.9)
Tubulointerstitial disease	11 (20)	7 (27.8)	4 (14.8)
FSGS/Alport disease	10 (22.2)	5 (27.8)	5 (18.5)
TMA/vascular disease	2 (4.4)	1 (5.5)	1 (3.7)
Nonspecific nondeterminant	6 (13.4)	3 (16.7)	3 (11.1)

CI, confidence interval; CKD, chronic kidney disease; Dx, diagnosis; eGFR, glomerular filtration rate; FSGS, focal segmental glomerulosclerosis; IKGP, the Irish Kidney Gene Project; IQR, interquartile range; KDIGO, Kidney Disease: Improving Global Outcomes; TMA, thrombotic microangiopathy.

a One hundred seventy-one families (217 individuals), comprised probands and their clinically affected relatives aged ≥ 60 years referred to the IKGP for genetic sequencing. Forty-eight additional individuals (31 families) were included, who were older affected family members related to the probands aged < 60 years.

b The age at which the participant experiences the first symptoms or the initial clinical presentation/evaluation.

c Kidney function at last follow-up. Progression to kidney failure indicates the initiation of chronic dialysis or preemptive kidney transplantation at last follow-up.

d The renal survival time was determined by calculating the median time for single-record kidney failure data (if it occurred), which indicted the duration of an enrolled patient's preserved kidney function.

e Chronic kidney disease (eGFR grades) as per the KDIGO guidelines.

f All available final pathological diagnoses in patients who had undergone a diagnostic native kidney biopsy before genetic sequencing was reevaluated, and the impact of the molecular diagnosis on the final clinical diagnosis was assessed.

### Diagnostic Yield of Genomic Testing

**Figure 2:**
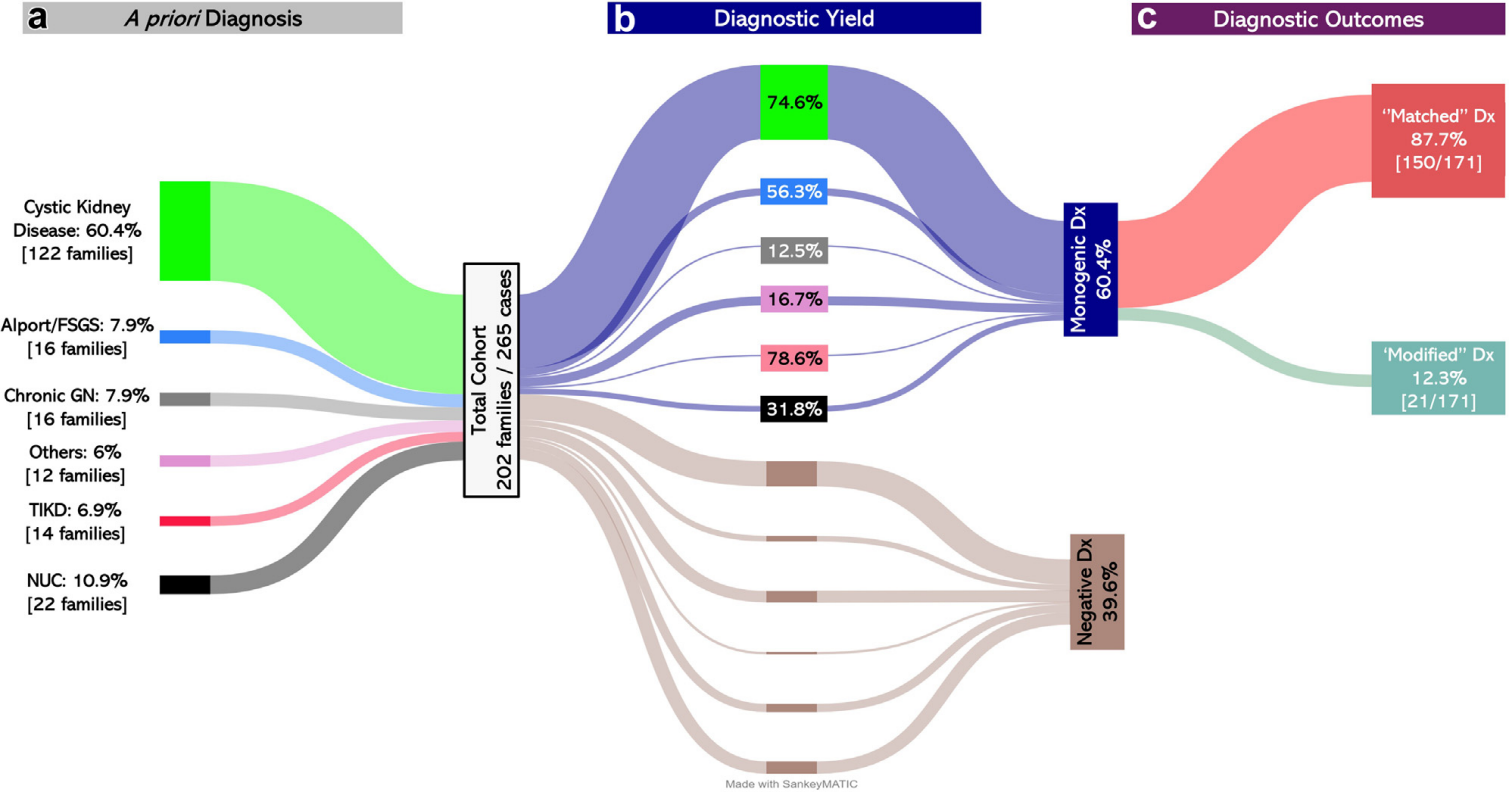
Distribution of the *a priori* diagnosis, diagnostic yield, and outcomes in the Irish Kidney Gene Project cohort of patients aged > 60 years. From the left side, nodes represent (a) the *a priori* diagnosis (the primary clinical presentation categories are displayed in color-coded patterns as follows: cystic kidney disorders [light green flow], Alport or focal segmental glomerulosclerosis [FSGS] [light blue flow], tubulointerstitial kidney disease [TIKD] [red flow], chronic glomerulonephritis [GN] [light gray flow], “Others” [pink flow], and families with nephropathy of undetermined cause [NUC] [black flow]); (b) the proportion of diagnostic yield (stratified by the *a priori* diagnosis), and (c) the genetic findings and their impact on the understanding of disease mechanisms, which were assessed at individual-level data. The width of the arrows is proportional to the number of patients. The Sankey diagram was created utilizing the SankeyMatic Tool. Dx, diagnosis

Most of the cohort underwent genetic sequencing using a single massive parallel sequencing technique (70.9%) (Figure 1). A small percentage (10.6%) underwent *MUC1* genotyping and MUC1-fs immunostaining, whereas 26.8% of the cohort had > 1 sequencing technique employed (Figure 1). As per the *a priori* diagnosis, the most common clinical diagnosis was cystic kidney disorders (60.4%) (Figure 2a). Other clinical presentations include Alport or FSGS (7.9%), tubulointerstitial kidney disease (6.9%), chronic GN (7.9%), and “Others” (6%) (Figure 2a). The prevalence of the *a priori* diagnosis varied according to age group categories (Supplementary Figure S1).

**Figure 3:**
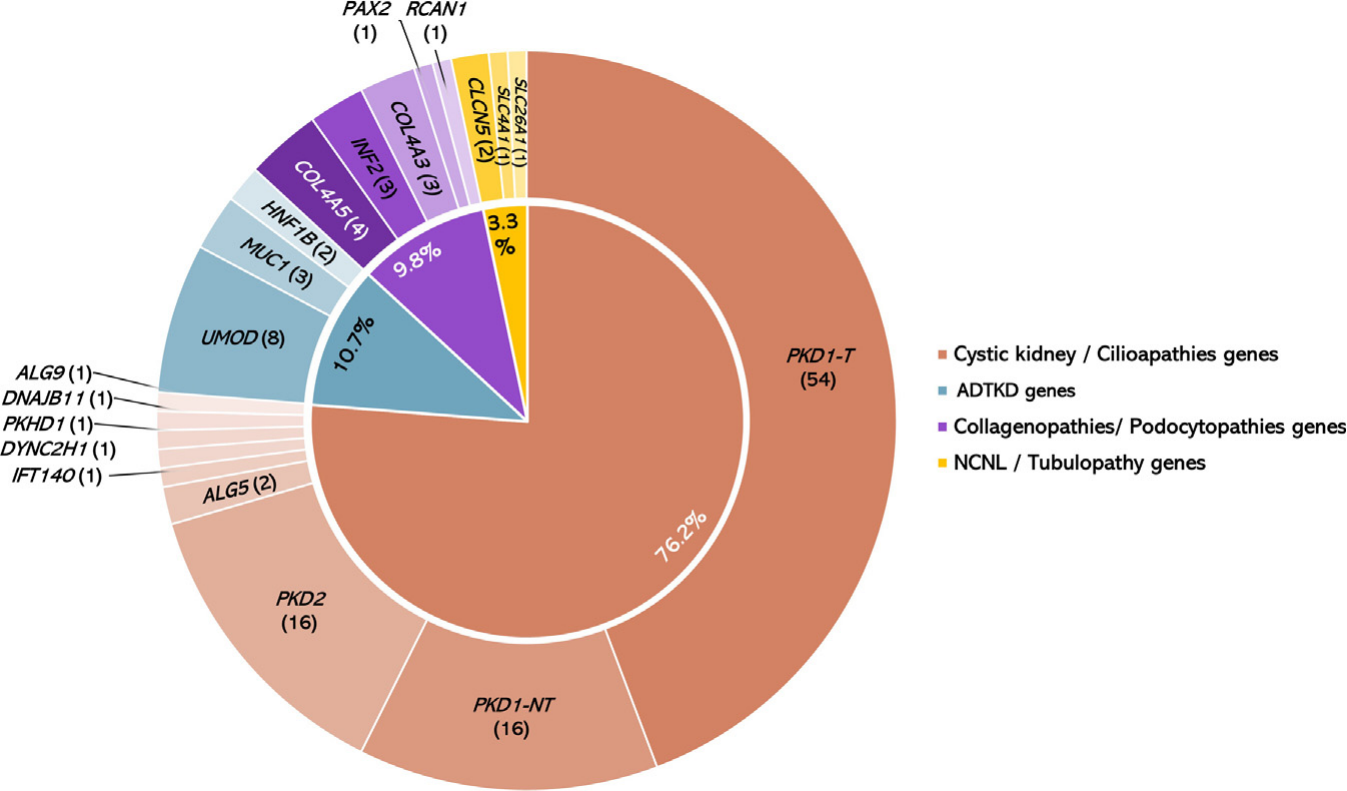
Genetic findings in patients aged > 60 years. The genetic results are illustrated as an inner pie containing the percentage of families according to their genetic group and an outer pie depicting the identified causative variants (variants per their genetic group) and the number of families in brackets. Color codes: variants in known cystic kidney or ciliopathies genes (tan), variants in autosomal dominant tubulointerstitial kidney disease (ADTKD)-causing genes (aqua), variants in collagenopathies and podocytopathies genes (purple); genetic variants causing nephrocalcinosis and nephrolithiasis (NCLC) and tubulopathy (gold). *n*, number; *PKD1*-NT, *PKD1* nontruncating variants (missense/in-frame variants); *PKD1*-T, PKD1 Truncating variants (frameshift, nonsense, splicing, and copy number variants).

We identified disease-causing variants in 60.4% of families (122/202) across 19 genes associated with a range of genetic kidney diseases (Figure 2b and Supplementary Figure S2A). The diagnostic yield was most frequently observed in older patients with cystic disorders (76.2%), tubulointerstitial kidney disease (10.7%), collagenopathies or podocytopathies (9.8%), and nephrolithiasis, nephrocalcinosis or tubulopathy (3.3%) (Figure 3 and Supplementary Table S1). Disease-causing variations within 5 genes accounted for 82.7% of all solved families as follows: *PKD1* (57.4%), *PKD2* (13.1%), *UMOD* (6.5%), and *COL4A*-related genes (*COL4A5* [3.3%] and *COL4A3* [2.4%]). The diagnostic genetic variants varied within each age group (Supplementary Figure S3A).

*PKD1* and *PKD2* disease-causing variants accounted for 70.5% of all positive genetic findings. About 30% of cystic kidney disease variants were found in patients aged > 75 years, including variants in 4 genes causing ADPKD-like disease spectrum (*ALG5*, *ALG9*, *DNAJB11*, and monoallelic *IFT140*) (Figure 3 and Supplementary Table S2). An additional family was identified to harbor biallelic pathogenic variations in *PKHD1* (F1271: a patient aged 62 years), which facilitated the refinement of the clinical diagnosis and allowed further phenotyping after confirming the molecular diagnosis.

The diagnostic yield among 80 families with noncystic kidney disease as an *a priori* diagnosis was 39% (Supplementary Figure S2B). Among families with noncystic kidney disease, the diagnostic yield among patients with an *a priori* diagnosis of tubulointerstitial kidney disease was 78.6%, 56.3% of families (*n* = 7) with Alport or FSGS, and only 1 family (12.5%) with *a priori* diagnosis of chronic GN was resolved (Figure 2b). Among 12 families referred with an *a priori* diagnosis of nephrolithiasis, nephrocalcinosis or tubulopathy, congenital anomalies of the kidney and urinary tract, collectively called “Others,” 16.7% were found to have a known monogenic disorder. An *a priori* diagnosis of “nephropathy of undetermined cause” accounted for 10.9% of families (Figure 2a). Of these, 7 families (31.8%) were identified to have a monogenic cause with disease-causing variants spanning over 7 genes (Supplementary Table S1). The diagnostic yield varied from 56% to 74% across different age groups, with no significant statistical difference reported between age groups (Supplementary Figure S3B and Table S3).

**Figure 4:**
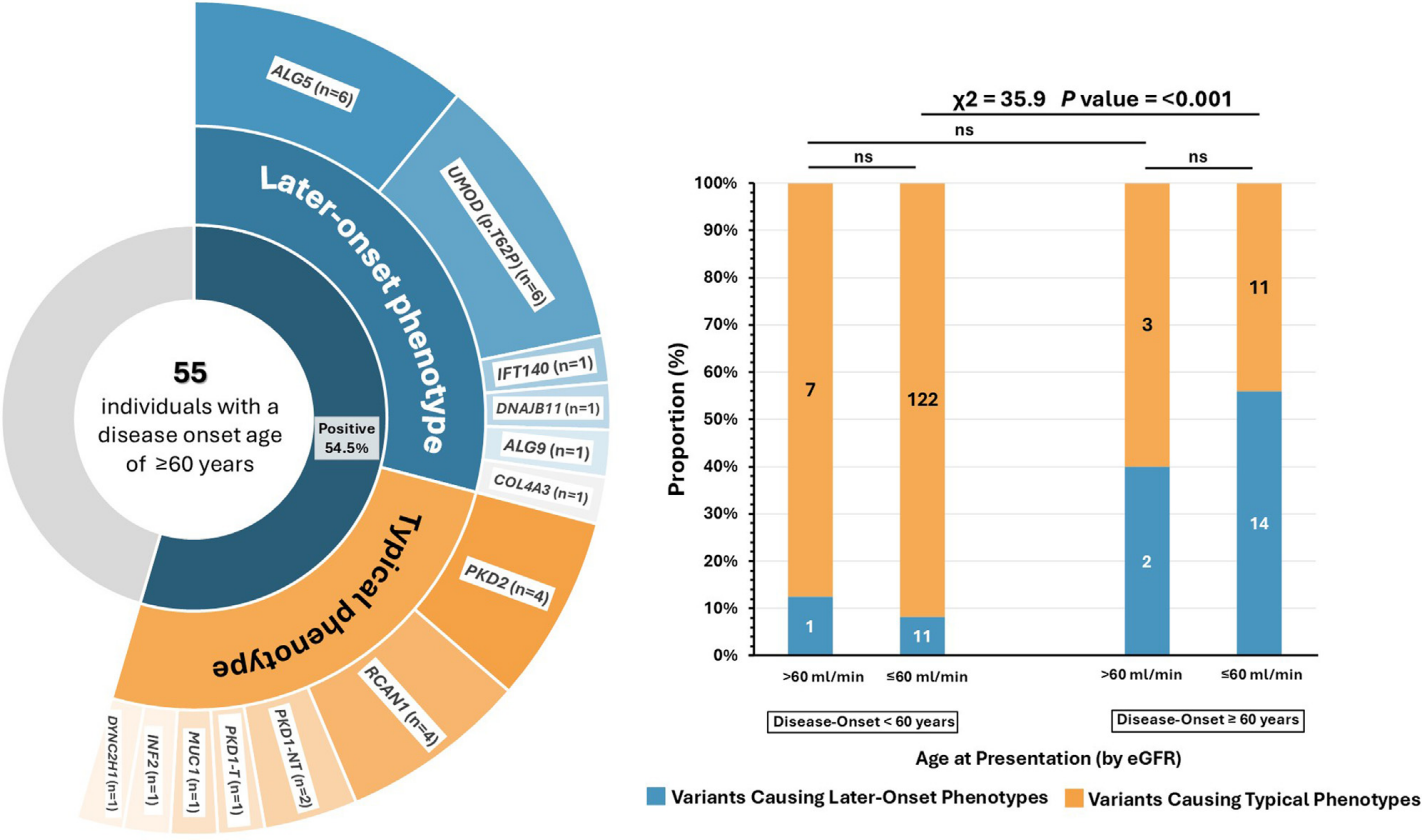
Genetic variants distribution in older patients based on disease-onset. (Left) The onset of disease has been split into 2 binary age groups: ≥ 60 years or < 60 years, according to the onset of symptoms or presentation to medical attention. Of 55 older patients, 54.5% with disease-onset at aged ≥ 60 years had disease-causing variants. Diagnostic variants causing a later-onset kidney disease phenotype comprised monoallelic *COL4A3,* ADPKD-like disease spectrum (including *ALG5, ALG9, DNAJB11*, and monoallelic *IFT140* variants), and the *UMOD* p.Thr62Pro variant. Twenty seven of the 55 individuals who were clinically diagnosed at aged ≥ 60 years had a non-ADPKD phenotype. Fifteen of 27 of non-ADPKD individuals (55.5%) were shown to have positive genetic findings. (Right) In terms of the genetic distribution of older adults with CKD (i.e., estimated glomerular filtration rate (eGFR) < 60 ml/min per 1.73 m^2^), individuals with onset of disease at age ≥ 60 years had more prevalent genetic variants causing a later-onset kidney disease phenotype compared with those with earlier onset (56% vs. 8.3%; *P ≤* 0.001) compared with those with onset of disease < 60 years. No significant difference (ns) was observed among individuals with an eGFR > 60 ml/min per 1.73 m^2^ or within age groups. ADPKD, autosomal dominant polycystic kidney disease; CKD, chronic kidney disease.

In terms of genetic findings, 6.6% of positive families (8/122) had *UMOD* disease-causing variants, with the *UMOD* p.(Thr62Pro) variant, associated with intermediate autosomal dominant tubulointerstitial kidney disease–like nephropathy spectrum, most prevalent in 7 families (Supplementary Table S1). *MUC1* and *HNF1B* disease-causing variants were identified in 3 and 2 families, respectively (Figure 3). Seven families (5.7%) harbored disease-causing variants linked to collagenopathies (2 of the 4 cases with *COL4A5* variants were females and 3 families with monoallelic *COL4A3* genes). Five additional families harbored variants linked to podocytopathies, namely *INF2*, *PAX2*, and *RCAN1* (Figure 3). In 55 older individuals with an age of disease onset ≥ 60 years, 54.5% (30/55) harbored disease-causing variants (Figure 4). Out of 27 patients with non-ADPKD phenotypes, 55.5% (15/27) carried disease-causing variants. Compared with older adults with CKD and disease-onset < 60 years, genetic variants associated with “later-onset” phenotypes were more prevalent in individuals with CKD and disease onset at age ≥ 60 years (56% vs. 8.3%; *P* ≤ 0.001) (Figure 4).

It was determined that genetic findings, at individual-level diagnosis, were concordant with the *a priori* diagnosis (i.e., matched) in 87.7% of cases with a genomic diagnosis, whereas in 12.3%, they were discordant, yielding new insights into the underlying disease mechanism (Figure 2c). For instance, in 38% of families with nephropathy of undetermined cause, genomic testing provided a precise molecular diagnosis, which, to that point, was unattainable. Furthermore, for variants associated with cystic kidney disease (*n* = 1), nephrocalcinosis and nephrolithiasis (*n* = 1), tubulointerstitial kidney disease (*n* = 3), and collagenopathies or podocytopathies (*n* = 5), our understanding of the disease process was modified as a result of the molecular diagnoses (Supplementary Table S4).

### Impact of Genetic Diagnosis on Clinical Management

Next, we evaluated the extent to which a molecular diagnosis led to a change in clinical management for each patient (Table 2). It was documented that 95.3% of genetic results (163/171) resulted in a change in the individual's treatment plan across 1 (or more) of 5 domains: cascade familial testing (73.7%), familial genetic counseling (54.4%), guiding in risk of disease progression (26.9%), modification of treatment plan (24%), and the facilitation of kidney transplant workup and follow-up (15.2%). Approximately one-third of the participants experienced a change in treatment management across > 2 domains (Table 2).

**Table 2:** Clinical impact attributable to the identification of monogenic nephropathy in patients aged > 60 years

(a) Distribution of clinical impact domains of individuals with disease-causing variant carriers					
Clinical impact domains	1 domain	2 domains	3 domains	4 domains	5 domains
% per individual (*n* = 163)	36.2% (59)	34.4% (56)	20.2% (33)	8% (13)	1.2% (2)
(b) Impact of genetic diagnosis on clinical management per *a priori* diagnosis					
*A priori* diagnosis (number of cases with disease-causing variants)	Cascade familial testing, *n* (%)	Kidney transplant impacts, *n* (%)	Genetic counseling, *n* (%)	Modifying medical treatment(s), *n* (%)	Prognostication, *n* (%)
Cystic kidney disease (polycystic kidney disease) (*n* = 129)	87 (67.4)	13 (10.1)	60 (46.5)	28 (21.7)	25 (19.4)
Alport / focal segmental glomerulosclerosis (*n* = 15)	15 (100)	15 (100)	15 (100)	15 (100)	15 (100)
Tubulointerstitial kidney disease (*n* = 11)	11 (100)	5 (45.5)	6 (54.5)	4 (36.4)	3 (27.3)
Chronic glomerulonephritis GN (*n* = 2)	2 (100)	0 (0)	2 (100)	0 (0)	2 (100)
Nephropathy of undetermined cause (*n* = 10)	8 (80)	3 (30)	10 (100)	0 (0)	3 (30)
Others (*n* = 4)	3 (75)	3 (75)	4 (100)	3 (75)	4 (100)
Total (*N* = 171)	126 (73.7)	26 (15.2)	93 (54.4)	41 (24)	46 (26.9)

(a) The frequency of clinical impact domains among individuals who tested positive for disease-causing variants, in which the clinical impact was evaluated among the following 5 domains: (i) cascade familial testing; (ii) kidney transplant impacts, defined as facilitating the evaluation and follow-up of kidney transplants, including donor assessment; (iii) referral to genetic counseling; (iv) modifying medical treatment(s), which include modifying of the individual's treatment plan by the introduction or cessation of medications, assessing extrarenal involvement; and (v) prognostication via determining the risk of disease progression. (b) Impact of genetic diagnosis on clinical management in 171 patients.

Next, we assessed the pathogenetic impact on patient clinical management (described in the Methods). Forty-five of the older cohort who underwent sequencing had previously undergone a kidney biopsy (the pathology information is presented in Table 1). Disease-causing variants were identified in 40% of cases (18/45) across all histopathological subgroups. Of kidney biopsies suggestive of nongenetic origin, 33.3% (6/18) have positive genetic diagnosis (Supplementary Figure S4). In 38.9% of cases (7/45), a genomic diagnosis changed the histopathological diagnosis, correcting the underlying disease mechanism in 4 cases and establishing a precise molecular diagnosis in 3 cases with nonspecific pathological findings, whereas 61.1% of histopathological diagnoses (11/18) were consistent with genetic findings (Supplementary Figure S4).

### Factors Associated With Obtaining Genetic Diagnosis and Disease Progression

Predictors of a genetic diagnosis were a family history of kidney disease and *a priori* diagnosis of cystic kidney disease, Alport or FSGS, and tubulointerstitial kidney disease (Table 3). We identified positive genetic findings in 67.9% of 159 families with a family history of kidney disease compared with 32.5% of 43 individuals without a family history (*P* = <0.001; Supplementary Figure S5). Males, early-onset disease, and chronic GN (compared with cystic kidney disease) were significant risk factors for kidney failure in Cox proportional hazard regression models (Supplementary Table S5). However, the presence of a genetic diagnosis was not significantly associated with an increased risk of kidney failure (hazard ratio: 1.29; 95% confidence interval: 0.95–1.74; *P* = 0.097). Individuals with variants causing “later-onset” phenotypes were associated with delayed progression to kidney failure (hazard ratio: 0.52; 95% confidence interval: 0.27–0.98; *P* = 0.043) (Supplementary Table S5).

**Table 3:** Factors associated with obtaining a genetic diagnosis

Variables	OR (95% CI)	*P*-value
Family history of CKD (yes)	6.85 (2.44–19.17)	<0.001
Age at disease onset (< 60 yrs)	1.19 (0.33–4.25)	0.783
Hypertension (yes)	2.36 (0.76–7.33)	0.136
Diabetes mellitus (yes)	0.73 (0.27–1.95)	0.537
Age at kidney failure (yrs)	1.001 (0.96–1.04)	0.941
Age group (yrs)		
60 to < 65 (vs. ≥ 80)	1.013 (0.16–6.21)	0.989
65 to < 70 (vs. ≥ 80)	0.83 (0.21–3.31)	0.8
70 to < 75 (vs. ≥ 80)	0.64 (0.17–2.38)	0.509
75 to < 80 (vs. ≥ 80)	0.39 (0.11–1.37)	0.142
Kidney failure at time of genetic testing (yes)	1.21 (0.72–2.03)	0.471
*A priori* diagnosis^a^		
CysKD (vs. GN)	26.06 (2.64–256.78)	0.005
Tubulointerstitial kidney disease (vs. GN)	64.54 (2.49–167.67)	0.012
Alport / FSGS (vs. GN)	12.16 (0.99–149.48)	0.049
NUC (vs. GN)	5.08 (0.43–59.83)	0.196
Others (vs. GN)	4.06 (0.25–64.98)	0.322

CI, confidence interval; CKD, chronic kidney disease; CysKD, cystic kidney disease; FSGS, focal segmental glomerulosclerosis; GN; glomerulonephritis; NUC, nephropathy of undetermined cause; OR, odds ratio.

Marginal logistic regression analysis employed the generalized estimating equation–population average model fitted to quantify the factors associated with obtaining a genetic diagnosis, which was adjusted for sex and familial clusters using family identifiers as a random variable.

a Referenced to chronic glomerulonephritis, which is the least *a priori* diagnosis with a diagnostic yield.

## Discussion

This study demonstrates the effectiveness and advantages of genetic testing in individuals suspected of having genetic kidney disease who are aged ≥ 60 years at the time of genetic testing. Disease-causing variants were detected in 60.4% of the families and one-third of families (39%) with noncystic kidney disease. Furthermore, our findings demonstrated that up to 1 in 10 patients had their *a priori* diagnosis modified based on the genetic findings. The results of this study also suggest that the effects of the results extend beyond diagnosis. For instance, results in 15% of solved cases facilitated kidney transplant workup, including donor selection, follow-up, diabetes management in pathogenic *HNF1B* variants, and primary disease recurrence.

In an aging population, clinical heterogeneity and declining kidney function with age may blur the lines of who should be tested. We demonstrated the enrichment of variants causing less-penetrant phenotypes in a cohort of older adults with suspected genetic kidney disease. This suggests that the role of monogenic impact is consistent across the lifespan. Although it is generally recognized that the investigation of genetic kidney disease is not precluded by age alone, the role of genomic testing in the diagnosis of patients with CKD must be supported, because the prevalence of kidney diseases is higher in an older population and identifying an accurate diagnosis of the underlying etiology is essential, regardless of age.^37^^,^^38^

In the largest study published to date of exome sequencing in patients with CKD, 29% of the 3315 patients sequenced were aged ≥ 65 years, of whom genetic findings had been identified at 15.9%.^11^ Despite a wide range of genetic findings, cystic kidney disease and *COL4A*-related conditions accounted for < 6% of the total in individuals aged ≥ 60 years,^11^ which is different from the results in our study (∼52%), highlighting that the variable gene frequencies across different populations, even across predominantly Caucasian populations, is evident; this may be attributed to differences in sample sizes, underlying phenotypes studied, and genotyping techniques applied. The use of diverse populations offers significant scientific advantages in analyzing genetic variant frequencies and their impact.

A recent study explored the genetic etiologies of CKD in more than 1000 individuals, using a broad gene panel of 382 Mendelian genes, of whom 26.5% of participants were aged ≥ 60 years.^39^ Positive genetic findings were identified in approximately 21.1% of the entire cohort. Notably, the diagnostic yield was 15.6% among individuals aged ≥ 60 years. Similarly, in another study of over 1600 patients sequenced via a broad gene panel of 385 genes, ∼24% of the cohort was aged ≥ 65 years.^40^ They demonstrated an overall frequency of identifying a genetic causative variant of 20.8%, whereas the frequency in those aged ≥ 65 years was 16.9%. Cases with monogenic causes were younger than those without a variant. Of note, this interesting observation is mainly derived from the increased proportion of solved cases in younger groups. Unlike the latter 2 studies,^39^^,^^40^ we found no significant difference in the diagnostic yield based on disease-onset, suggesting that molecular genetic diagnosis can be made at any stage, which may benefit patients and their families. We had a higher diagnostic yield than previous studies,^11^^,^^39^^,^^40^ suggesting that highly selected groups at higher risk of genetic kidney disease were overrepresented (increased cystic kidney disease, family history clustering, and various sequencing techniques). Therefore, harmonizing the age cutoff and other criteria that determine the underlying genetic phenotypes and studied patient characteristics is essential to draw meaningful conclusions about disease outcomes and prevalence estimates across studies.

In our study, molecular diagnosis was established, on average, 25 years after the initial presentation, which may have increased the diagnostic yield by providing additional time for phenotype penetrance and familial cascade clinical utility. Genetic diagnosis at a younger age allows for earlier intervention; however, for more slowly progressive kidney diseases, probands may be older or have reached kidney failure before other family members develop CKD.

In older individuals with CKD, these data recognize certain traits critical to their clinical evaluation. Family history of kidney disease and *a priori* diagnoses of cystic kidney disease, Alport or FSGS, and tubulointerstitial kidney disease were the most consistent associations with monogenic kidney disorders. Patients with positive genetic findings had no increased risk of kidney failure (Supplementary Table S5), contrary to a recent study.^41^ Nevertheless, this particular point will require further investigation in larger cohorts. In addition, our results suggest that genetic variants associated with less-penetrant phenotypes have been detected predominantly in individuals with a disease onset age ≥ 60 years (∼20% of the total cohort) (Figure 4). These include ADPKD-like variants, monoallelic *COL4A3* variants, and the *UMOD* p.Thr62Pro variant. The *UMOD* p.Thr62Pro variant, for example, has been recently identified as a variant that may infer a reduced severity in autosomal dominant tubulointerstitial kidney disease despite its relatively high prevalence in control populations^42^ (the clinical characteristics are presented in Supplementary Table S6). By demonstrating the enrichment of genetic variants associated with delayed onset of kidney failure in older patients with CKD, we show that the underlying disease may rather be steadily progressive, with minimal nonspecific features in the initial phases. Therefore, by reducing the time necessary to make a diagnosis, genetic testing can broaden the intervention window of medications or lifestyle changes that protect before the onset of progressive CKD, which may lead to irreversible changes eventually, and also may enable prospective enrolment in therapeutic studies.^37^^,^^38^ Altogether, these observations highlight the relevance of genetic testing in older adults with CKD and kidney failure, and age should not be a deterrent.

Our results also show that genetic testing offers several benefits to older individuals. Two-thirds of individuals with positive findings had a relative benefit from genetic testing (∼74% cascade familial testing), and 1 in 2 individuals underwent dedicated familial genetic counseling about their genetic findings. Although this cohort molecular diagnosis was made 25 years after disease onset, incorporating cost-effectiveness into resource-intensive genetic tests may be beneficial. The cost-effectiveness of early genetic testing was demonstrated in a study involving approximately 500 individuals with CKD, along with a 67% diagnostic rate.^43^ In addition, they showed beneficial impact on various clinical management domains, similar to our cohort, such as cascade testing (23.2 vs. 68.4%), informing disease progression (28.2 vs. 30.1%), modification of treatment plan (29.2 vs. 24.8%), and kidney transplant workup (11.9 vs. 18.1%). Therefore, early resolution of the diagnostic odyssey in older adults with CKD may lead to extended cost savings.

Our study has several limitations. The inclusion of a relatively small number of patients, a small number of people diagnosed with CKD after the age of 60 years, and the absence of a control group may not accurately reflect the real-world prevalence of genetic kidney disease in older adults. This may have contributed to an enrolment bias toward highly selected groups because of the nature of registry data. As a single-center study with predominantly Caucasian patients, the extent to which the results can be generalized to other ancestries is unclear. In addition, reanalysis of sequencing data or the use of different sequencing methods can address families with nondiagnostic variants.^44^ Therefore, prospective population-based multicenter studies are required to address these points.

In conclusion, we identified disease-causing variants in up to 60% of patients with CKD aged ≥ 60 years, with a predominance of variants associated with less-penetrant phenotypes. Monogenic kidney disease awareness improves family counseling and personalized medical treatment in this age group.

## Disclosure

EAEE reports funds from the Royal College of Surgeons in Ireland StAR PhD. GLC reports receipt of research funding from BioMarin. PJC reports HRB research grants and funding from BioMarin Pharmaceutical. MDG reports the following: grants and scientific funding from the Science Foundation Ireland (Grant number: 3/RC/2073_P2); Orbsen Therapeutics Ltd., Galway; and the European Commission; honoraria from the American Society of Nephrology and Mayo Clinic; and travel support from Mayo Clinic, Florida. AJBS reports royalties from UpToDate; honoraria from Natera, Inc.; and advisory or leadership role with Natera, Inc. All the other authors declared no competing interests.

## Supplementary Material

Supplementary File (PDF) **Figure S1.** Proportion of *a priori* diagnosis according to the age groups. **Figure S2.** Mode of inheritance of identified genes of whole cohort and the diagnostic yield among families with noncystic clinical diagnosis. **Figure S3.** (A) Genetic findings and (B) diagnostic yield patients aged > 60 years with suspected genetic kidney disease based on various age groups. **Figure S4.** Diagrammatic illustration summarizes the histopathological dominant lesions and the outcomes of the analytical workflow for the cohort that underwent native kidney biopsy. **Figure S5.** Diagnostic yield based on the presence of family history. **Table S1.** Genetic variations reported in this cohort. **Table S2.** Genetic variants distribution in patients aged > 60 years. **Table S3.** Diagnostic yield based on age groups and age at disease onset. **Table S4.** Patients with modified diagnosis. **Table S5.** Kidney survival predictors in older adults with suspected genetic kidney disease. **Table S6.** Phenotypes of *UMOD* p.T62P carriers.
