# Supplementary material for: Characterization of Monogenic Kidney Disease in Older Patients With CKD
Source: Kidney Int Rep. 2025 Apr 22;10(7):2140–52. doi: 10.1016/j.ekir.2025.04.017 (PMC12266177; doi:10.1016/j.ekir.2025.04.017)
Supplement: Supplementary File (PDF) — Figure S1. Proportion of a priori diagnosis according to the age groups. Figure S2. Mode of inheritance of identified genes of whole cohort and the diagnostic yield among families with noncystic clinical diagnosis. Figure S3. (A) Genetic findings and (B) diagnostic yield patients aged > 60 years with suspected genetic kidney disease based on various age groups. Figure S4. Diagrammatic illustration summarizes the histopathological dominant lesions and the outcomes of the analytical workflow for the cohort that underwent native kidney biopsy. Figure S5. Diagnostic yield based on the presence of family history. Table S1. Genetic variations reported in this cohort. Table S2. Genetic variants distribution in patients aged > 60 years. Table S3. Diagnostic yield based on age groups and age at disease onset. Table S4. Patients with modified diagnosis. Table S5. Kidney survival predictors in older adults with suspected genetic kidney disease. Table S6. Phenotypes of UMOD p.T62P carriers. [file mmc1.pdf]

SUPPLEMENTARY FIGURES AND TABLES

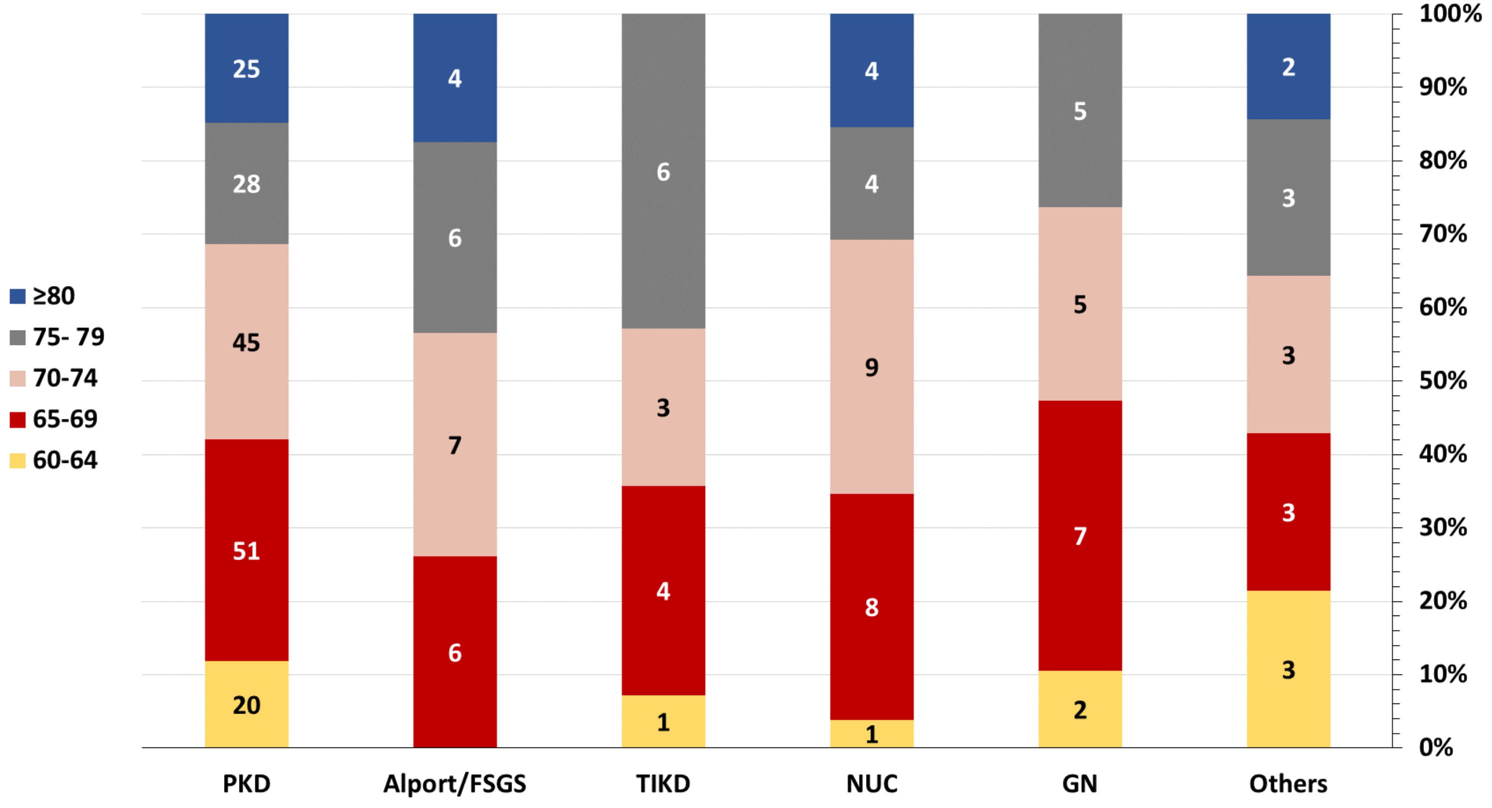

Figure S1| Proportion of the *a priori* diagnosis According to the Age Groups.

FSGS: focal segmental glomerulosclerosis; GN; glomerulonephritis; NUC: Nephropathy of undetermined cause; PKD: cystic kidney disease; TIKD: tubulointerstitial kidney disease

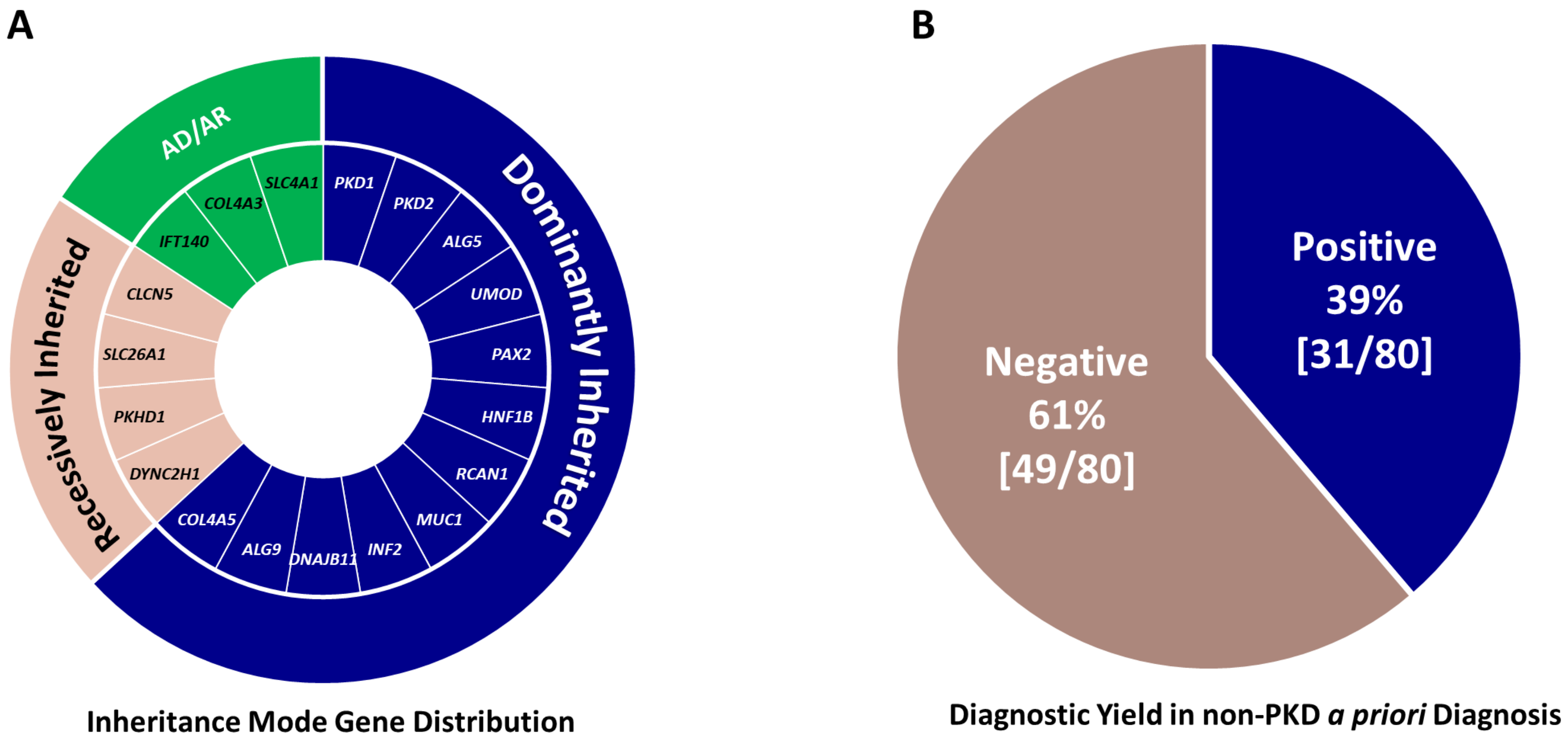

Figure S2 | Mode of Inheritance of Identified Genes of Whole Cohort and the Diagnostic Yield Among Families with Non-Cystic Clinical Diagnosis

AD: Autosomal dominant; AR: autosomal recessive; PKD: polycystic kidney disease

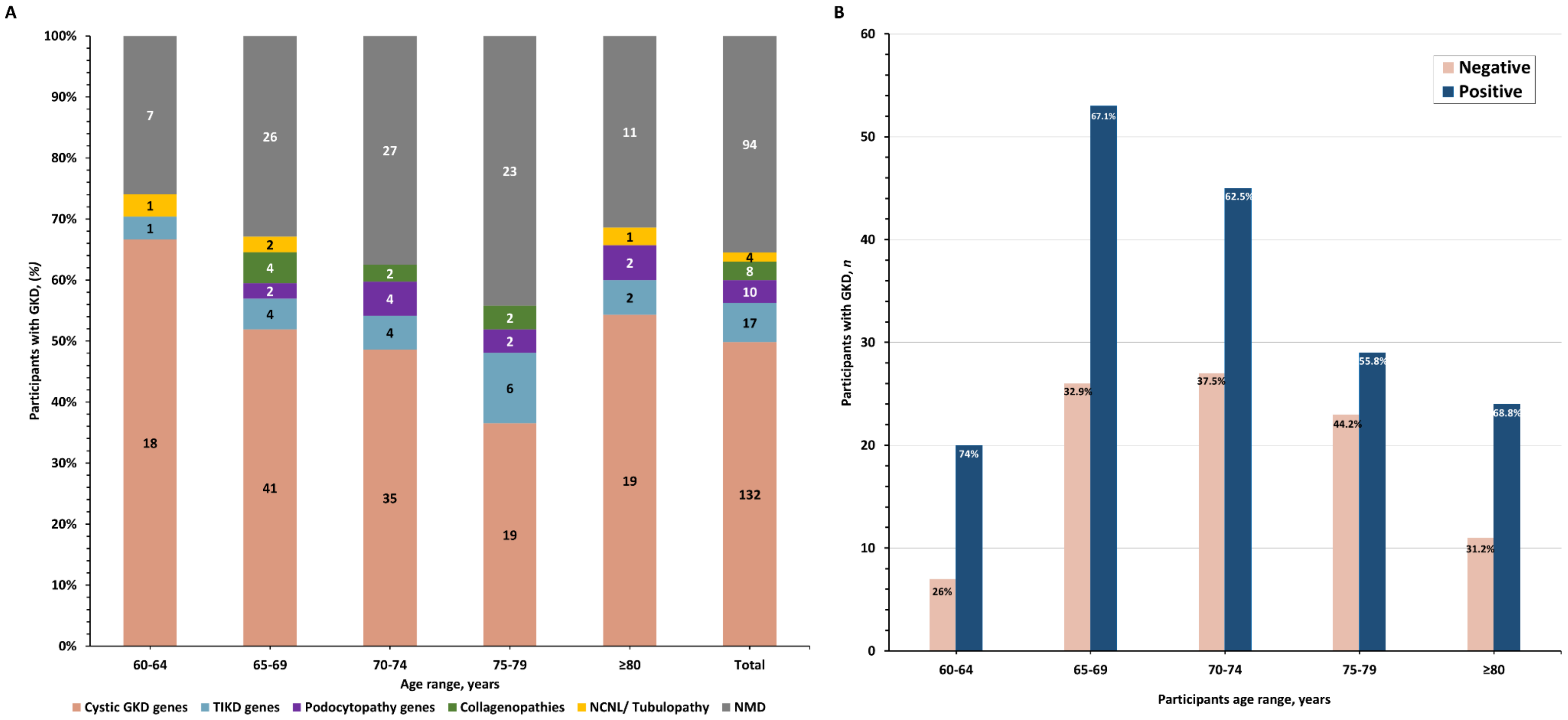

Figure S3 | (A) Genetic findings and (B) Diagnostic Yield Patients Over 60 Years of Age with Suspected Genetic Kidney Disease based on various age groups.

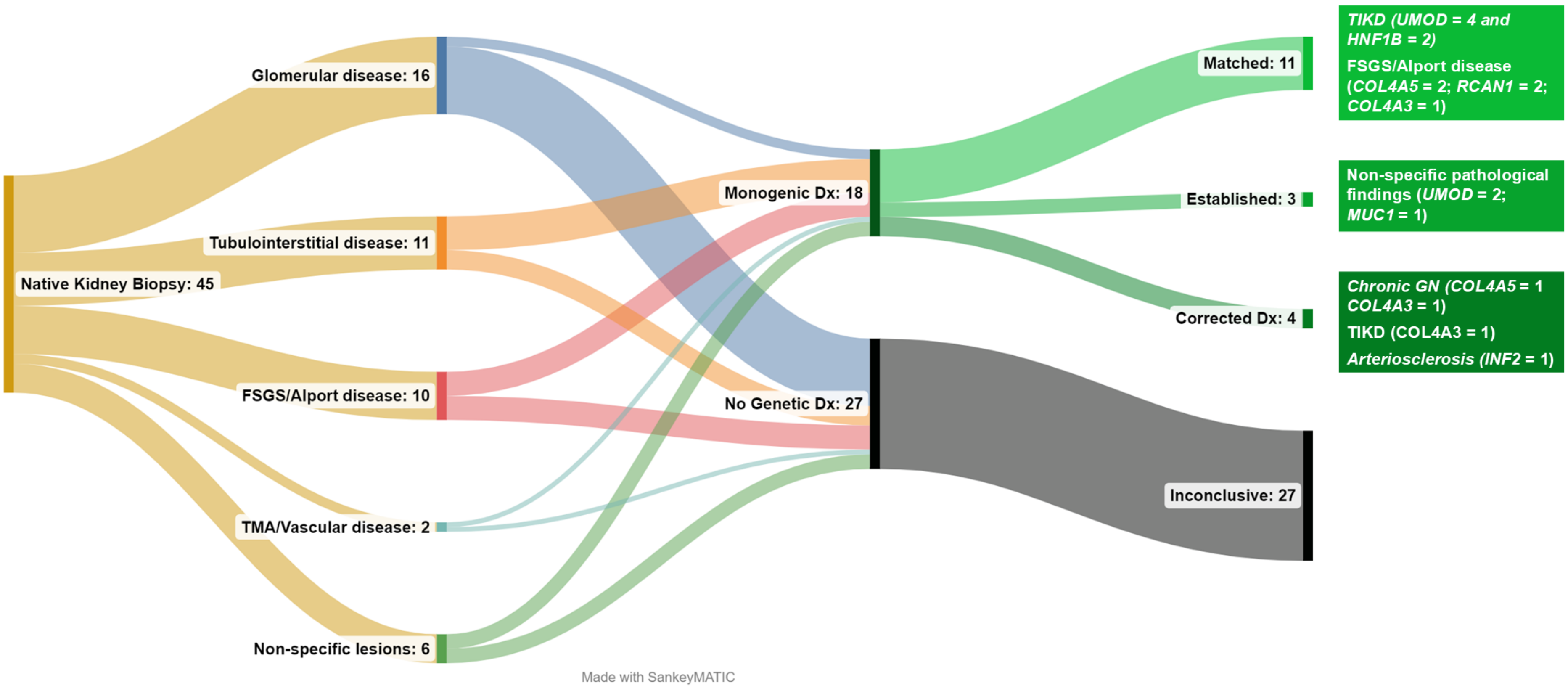

Figure S4 | Diagramtic illustration summarizes the histopathologically dominant lesions and the outcomes of the analytical workflow for the cohort that underwent native kidney biopsy. From the left side, nodes represent (i) the primary histological categories, (ii) the identification of variants with or without genetic testing, and (iii) the genetic findings and their impact on the understanding of disease mechanisms in those patients who underwent native kidney biopsy prior to genetic testing. The width of the arrows is proportional to the number of patients.). Of 39 kidney biopsies available for analysis, 16 biopsies were suggesting of non-genetic cause. These biopsies were in majority represent histological changes related to arteriosclerosis presumed of hypertension changes (n=1), chronic glomerular changes (glomerular basement membrane thickness without endotheliosis, patchy podocyte effacement, scattered tubulointerstitial changes with-/without tubulitis, and weak IgA deposits) (n=10), and none specific nodular or global glomerulosclerosis (n= 5). Positive genetic diagnosis has been identified in 37.5% [6/16] of patients with kidney biopsies suggestive of non-genetic disease. The Sankey diagram was created utilizing the SankeyMatic Tool. Dx: diagnosis; FSGS: focal segmental glomerulosclerosis; GN; glomerulonephritis; TIKD: tubulointerstitial kidney disease; TMA: thrombotic microangiopathy

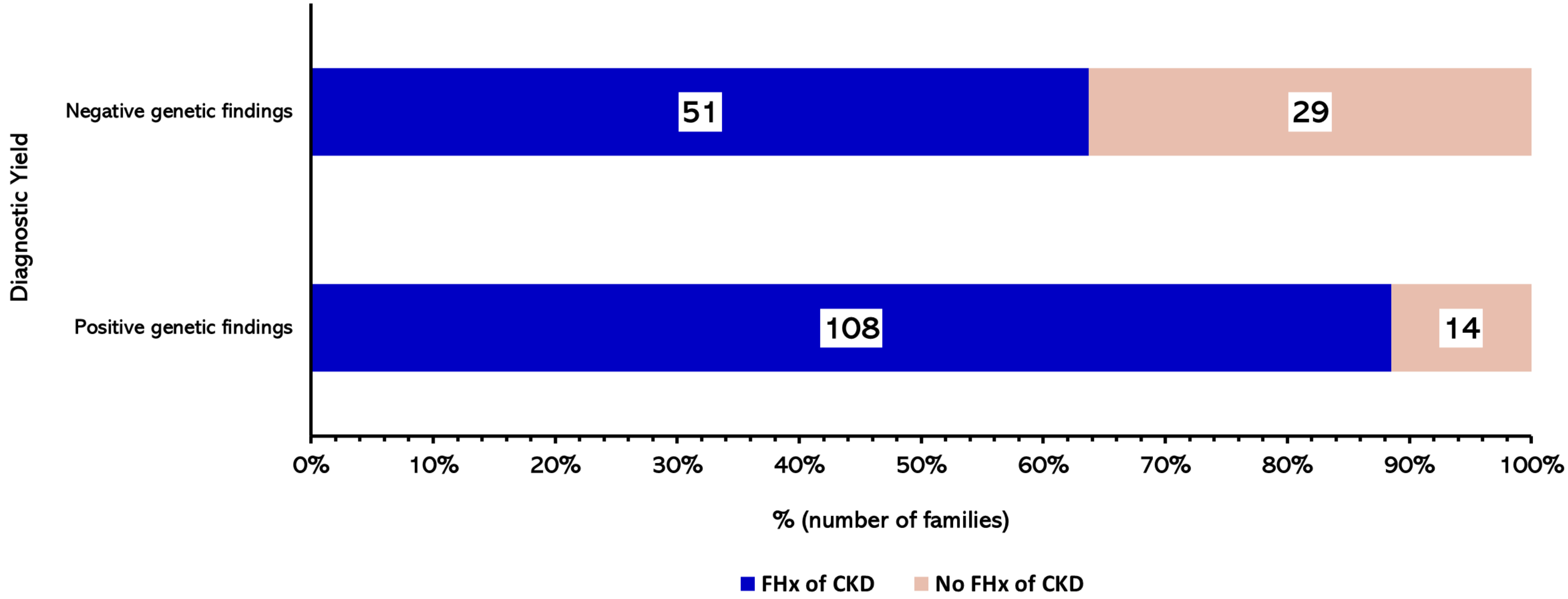

Figure S5 | Diagnostic Yield Based on the Presence of Family History

CKD: chronic kidney disease; FHx: family history

Table S1 | Genetic Variations Reported in this Cohort

Family ID	The *a priori* diagnosis category	Gene	# OMIM and Mode of Inheritance	Variant Type (Exon)	Coding Alteration (Zygosity)	AF (gnomAD)	ACMG Classification (Evidence)	Individuals Sequenced (*n*)
**Cystic Kidney and Ciliopathies Genes (Transcripts: *PKD1* (NM_001009944.3), *PKD2* (NM_000294.4), *IFT140* (NM_014714.4), *PKHD1* (NM_138694.4), *ALG5* (NM_013338.5), *ALG9* (NM_024740.2), *DNAJB11* (NM_016306.6), *DYNC2H1* (NM_001377.3))**								
**F1031**	CysKD	*PKD1*	# 601313 (AD)	Frameshift deletion (Ex. 15)	c.5843_5844del p.V1948Efs*41 (het.)	0	LP (PVS1, PM2)	2
**F1045**	CysKD	*PKD1*	# 601313 (AD)	Stopgain (Ex. 21)	c.8008C>T p.Q2670Ter (het.)	0	P (PVS1, PM2, PP5)	1
**F1108**	CysKD	*PKD1*	# 601313 (AD)	Frameshift deletion (Ex. 11)	c.2396_2421del p.R799fs (het.)	0.0000006962	LP (PVS1, PM2)	1
**F1119**	CysKD	*PKD1*	# 601313 (AD)	Frameshift deletion (Ex. 15)	c.5014_5015del p.R1672Gfs*98 (het.)	0.000003726	LP (PVS1, PM2)	1
**F151**	CysKD	*PKD1*	# 601313 (AD)	In-frame deletion (Ex. 18)	c.7303_7317del p.2435_2439del (het.)	0	LP (PM1_Supp, PM2, PM4, PP3)	1
**F19**	CysKD	*PKD1*	# 601313 (AD)	Splicing (Ex. 3)	c.288-2A>G p.? (het.)	0	P (PVS1, PM2, PP5)	2
**F24**	CysKD	*PKD1*	# 601313 (AD)	Splicing (Ex. 16)	c.6919-9G>GA p.? (het.)	0	P (PVS1, PM2, PP3)	2
**F332**	CysKD	*PKD1*	# 601313 (AD)	Frameshift deletion (Ex. 29)	c.9736delC p.R3246fs (het.)	0	LP (PVS1, PM2)	1
**F333**	CysKD	*PKD1*	# 601313 (AD)	Stopgain (Ex. 41)	c.11525G>A p.W3842Ter (het.)	0	P (PVS1, PM2, PP5)	1
**F336**	CysKD	*PKD1*	# 601313 (AD)	CNV (Ex. 27 to 30)	Multi-exon deletion (het.)	0	P (2A, 4L)	1
**F339**	CysKD	*PKD1*	# 601313 (AD)	Frameshift deletion (Ex. 19)	c.7495delC p.H2499fs (het.)	0	LP (PVS1, PM2)	1
**F341**	CysKD	*PKD1*	# 601313 (AD)	Stopgain (Ex. 11)	c.2534T>A p.L845Ter (het.)	0	LP (PVS1, PM2)	2
**F346**	CysKD	*PKD1*	# 601313 (AD)	Splicing (Ex. 13)	c.2986-1G>A p.? (het.)	0	P (PVS1, PM2, PP3)	1
**F355**	CysKD	*PKD1*	# 601313 (AD)	Splicing (Ex. 10)	c.2097+5C>T p.? (het.)	0	P (PVS1, PM2, PP3, PP1_mod)	2
**F357**	CysKD	*PKD1*	# 601313 (AD)	Frameshift insertion (Ex. 5)	c.1171dupA p.I391fs (het.)	0	LP (PVS1, PM2)	1
**F358**	CysKD	*PKD1*	# 601313 (AD)	Frameshift deletion (Ex. 26)	c.9292delC p.L3098Wfs*218 (het.)	0	LP (PVS1, PM2)	1
**F360**	CysKD	*PKD1*	# 601313 (AD)	Frameshift deletion (Ex. 15)	c.5142delC p.F1714fs (het.)	0	LP (PVS1, PM2)	1
**F366**	CysKD	*PKD1*	# 601313 (AD)	Splicing (Ex. 41)	c.11411+1G>C p.? (het.)	0	P (PVS1, PM2, PP3)	1
**F367**	CysKD	*PKD1*	# 601313 (AD)	Frameshift deletion (Ex. 41)	c.11454delC p.G3818fs (het.)	0	P (PVS1, PM2, PP5)	1
**F370**	CysKD	*PKD1*	# 601313 (AD)	Frameshift deletion (Ex. 3)	c.348_352del p.N116fs (het.)	0	P (PVS1, PM2, PP5)	1
**F371**	CysKD	*PKD1*	# 601313 (AD)	Stopgain (Ex. 1)	c.99C>CA p.C33Ter (het.)	0	LP (PVS1, PM2)	1

**F375**	CysKD	*PKD1*	# 601313 (AD)	Frameshift deletion (Ex. 15)	c.4654delG<br>p.V1552fs (het.)	0	P (PVS1, PM2, PP5)	1
**F376**	CysKD	*PKD1*	# 601313 (AD)	Frameshift insertion (Ex. 40)	c.11312_11313insTC<br>p.S3771fs (het.)	0	LP (PVS1, PM2)	2
**F380**	CysKD	*PKD1*	# 601313 (AD)	Frameshift deletion (Ex. 15)	c.4782delT<br>p.G1594fs (het.)	0	LP (PVS1, PM2)	1
**F383**	CysKD	*PKD1*	# 601313 (AD)	Stopgain (Ex. 20)	c.7833C>G<br>p.Y2611Ter (het.)	0	P (PVS1, PM2, PP5)	1
**F385**	CysKD	*PKD1*	# 601313 (AD)	Stopgain (Ex. 18)	c.7369G>T<br>p.E2457Ter (het.)	0	LP (PVS1, PM2)	1
**F391**	CysKD	*PKD1*	# 601313 (AD)	Missense (Ex. 9)	c.1831C>T<br>p.R611W (het.)	0	LP (PS4, PM2, PP3, PP5)	2
**F393**	CysKD	*PKD1*	# 601313 (AD)	Splicing (Ex. 38)	c.11017-10C>A<br>p.? (het.)	0	P (PVS1, PM2, PP5_mod)	1
**F394**	CysKD	*PKD1*	# 601313 (AD)	Stopgain (Ex. 15)	c.6367C>T<br>p.Q2123Ter (het.)	0	P (PVS1, PM2, PP5)	1
**F401**	CysKD	*PKD1*	# 601313 (AD)	Frameshift insertion (Ex. 42)	c.11699_11700insCTCTCGCTGCC<br>p.P3900fs (het.)	0	LP (PVS1, PM2, P1_supp)	3
**F439**	CysKD	*PKD1*	# 601313 (AD)	Splicing (Ex. 7)	c.1606+5G>GA<br>p.? (het.)	0	LP (PVS1, PM2)	1
**F441**	CysKD	*PKD1*	# 601313 (AD)	Stopgain (Ex. 21)	c.8008C>T<br>p.Q2670Ter (het.)	0	P (PVS1, PM2, PP5)	1
**F443**	CysKD	*PKD1*	# 601313 (AD)	Stopgain (Ex. 22)	c.8056C>T<br>p.Q2686Ter (het.)	0	LP (PVS1, PM2)	1
**F462**	CysKD	*PKD1*	# 601313 (AD)	In-frame deletion (Ex. 15)	c.3719_3721del<br>p.1240_1241del (het.)	0	LP (PM2, PM4, PS4_Mod, PP5)	1
**F466**	CysKD	*PKD1*	# 601313 (AD)	Frameshift deletion (Ex. 10)	c.2032delG<br>p.A678fs (het.)	0	LP (PVS1, PM2)	1
**F5**	CysKD	*PKD1*	# 601313 (AD)	Frameshift deletion (Ex. 46)	c.12604_12631del<br>p.Gly4202Sfs (het.)	0	P (PVS1, PM2, PP5)	1
**F522**	CysKD	*PKD1*	# 601313 (AD)	Frameshift deletion (Ex. 15)	c.5843_5844del<br>p.V1948Efs*41 (het.)	0	LP (PVS1, PM2)	3
**F530**	CysKD	*PKD1*	# 601313 (AD)	Splicing (Ex. 3)	c.288-2A>G<br>p.? (het.)	0	P (PVS1, PM2, PP5)	2
**F531**	CysKD	*PKD1*	# 601313 (AD)	Frameshift insertion (Ex. 44)	c.12039_12040insAC<br>p.V4014fs (het.)	0	LP (PVS1, PM2)	1
**F562**	CysKD	*PKD1*	# 601313 (AD)	Stopgain (Ex. 15)	c.4551C>G<br>p.Y1517Ter (het.)	0.000001372	P (PVS1, PM2, PP5)	2
**F567**	CysKD	*PKD1*	# 601313 (AD)	Stopgain (Ex. 15)	c.4306C>T<br>p.R1436Ter (het.)	0	P (PVS1, PM2, PP5)	1
**F574**	CysKD	*PKD1*	# 601313 (AD)	Stopgain (Ex. 15)	c.3972C>A<br>p.Y1324Ter (het.)	0	LP (PVS1, PM2)	1
**F611**	CysKD	*PKD1*	# 601313 (AD)	Stopgain (Ex. 46)	c.12673C>T<br>p.Q4225Ter (het.)	0	P (PVS1, PM2, PP5)	1
**F618**	CysKD	*PKD1*	# 601313 (AD)	In-frame deletion (Ex. 5)	c.858_932del<br>p.Gly287_Ser311del (het.)	0	LP (PM1, PM2, PM4)	1
**F623**	CysKD	*PKD1*	# 601313 (AD)	Stopgain (Ex. 40)	c.11343C>G<br>p.Y3781Ter (het.)	0	P (PVS1, PM2, PP5)	1
**F627**	CysKD	*PKD1*	# 601313 (AD)	Frameshift Insertion (Ex. 11)	c.2102_2103insAC<br>p.T702fs (het.)	0	LP (PVS1, PM2)	1

**F637**	CysKD	*PKD1*	# 601313 (AD)	Frameshift deletion (Ex. 15)	c.5014_5015del<br>p.R1672Gfs*98 (het.)	0.000003726	LP (PVS1, PM2)	1
**F650**	CysKD	*PKD1*	# 601313 (AD)	Stopgain (Ex. 21)	c.8008C>T<br>p.Q2670Ter (het.)	0	P (PVS1, PM2, PP5)	1
**F653**	CysKD	*PKD1*	# 601313 (AD)	In-frame deletion (Ex. 15)	c.3699_3701del<br>pA1233del (het.)	0	LP (PM1, PM2, PM4)	1
**F658**	CysKD	*PKD1*	# 601313 (AD)	Frameshift deletion (Ex. 15)	c.4452delG<br>p.Y1485Tfs*49 (het.)	0	LP (PVS1, PM2)	3
**F665**	CysKD	*PKD1*	# 601313 (AD)	Frameshift deletion (Ex. 15)	c.4452delG<br>p.Y1485Tfs*49 (het.)	0	LP (PVS1, PM2)	1
**F680**	CysKD	*PKD1*	# 601313 (AD)	CNV (Ex. 2 to 46)	Multi-exon deletion (het.)	0	P (2A, 4L)	1
**F685**	CysKD	*PKD1*	# 601313 (AD)	Frameshift deletion (Ex. 10)	c.2085delC<br>p.P695fs (het.)	0	P (PVS1, PM2, PP5)	1
**F688**	CysKD	*PKD1*	# 601313 (AD)	CNV (Ex. 3 to 11)	Multi-exon deletion (het.)	0	P (2A, 4L)	1
**F8**	CysKD	*PKD1*	# 601313 (AD)	Stopgain (Ex. 15)	c.6493C>T<br>p.Q2165Ter (het.)	0	P (PVS1, PM2, PP5)	1
**F894**	CysKD	*PKD1*	# 601313 (AD)	Frameshift Insertion (Ex. 23)	c.8371dupC<br>p.R2791Pfs*31 (het.)	0	LP (PVS1, PM2)	1
**TF88**	CysKD	*PKD1*	# 601313 (AD)	Stopgain (Ex. 4)	c.430C>T<br>p.Q144Ter (het.)	0	LP (PVS1, PM2)	1
**TF90**	CysKD	*PKD1*	# 601313 (AD)	Frameshift insertion (Ex. 5)	c.912_913insG<br>p.Trp305Valfs*66 (het.)	0	LP (PVS1, PM2)	1
**F648**	CysKD	*PKD1*	# 601313 (AD)	Frameshift deletion (Ex. 43)	c.11936_11939del: p.Gln3979Argfs*4<br>c.11932del: p.Asp3978Thrfs*6 (Comp. het.)	0<br>0	LP (PVS1, PM2)<br>LP (PVS1, PM2)	1
**F603**	CysKD	*PKD1*	# 601313 (AD)	Missense (Ex. 37)	c.10942C>A<br>p.P3648T (het.)	0	LP (PM1, PM2, PP3)	1
**F568**	CysKD	*PKD1*	# 601313 (AD)	Missense (Ex. 18)	c.7300C>T<br>p.R2434W (het.)	0.0000006918	LP (PM1, PM5, PP1_supp, PP3, PP5)	3
**F504**	CysKD	*PKD1*	# 601313 (AD)	Missense (Ex. 6)	c.1237T>G<br>p.F413V (het.)	0	LP (PM1, PM2, PP1_mod)	4
**F20**	CysKD	*PKD1*	# 601313 (AD)	Missense (Ex. 39)	c.11249G>A<br>p.R3750Q (het.)	0.0000006858	LP (PM1_supp, PM2, PM5, PP5, PP3)	2
**F1095**	CysKD	*PKD1*	# 601313 (AD)	Missense (Ex. 11)	c.2534T>C<br>p.L845S (het.)	0.000004847	P (PS1, PS4, PM2, PP3, PP5)	1
**F11**	CysKD	*PKD1*	# 601313 (AD)	Missense (Ex. 25)	c.9143T>G<br>p.L3048R (het.)	0	LP (PM2, PP1, PP3_strong)	3
**F1118**	CysKD	*PKD1*	# 601313 (AD)	Missense (Ex. 37)	c.10946C>A<br>p.P3649H (het.)	0	LP (PM1_supp, PM2, PM5, PP1)	2
**F115**	CysKD	*PKD1*	# 601313 (AD)	Missense (Ex. 39)	c.11249G>A<br>p.R3750Q (het.)	0.0000006858	LP (PM1_supp, PM2, PM5, PP5, PP3)	2
**F153**	CysKD	*PKD1*	# 601313 (AD)	Missense (Ex. 27)	c.9425T>C<br>p.L3142P (het.)	0	LP (PM2, PP1_mod, PP3, PP5)	3
**F287**	CysKD	*PKD1*	# 601313 (AD)	Missense (Ex. 15)	c.4082T>C<br>p.L1361P (het.)	0	LP (PM2, PP1_mod, PP3, PP5)	3
**F729**	CysKD	*PKD1*	# 601313 (AD)	Missense	nonsynonymous SNV -LR-PCR	-	-	1
**F143**	CysKD	*PKD2*	# 613095 (AD)	Frameshift insertion (Ex. 1)	c.195_196insGACC p.R65fs (het.)	0	LP (PVS1, PM2)	1
**F726**	CysKD	*PKD2*	# 613095 (AD)	Frameshift deletion (Ex. 1)	c.405delC p.G135fs (het.)	0	LP (PVS1, PM2)	1
**F573**	CysKD	*PKD2*	# 613095 (AD)	Frameshift deletion (Ex. 7)	c.1551delG p.L517fs (het.)	0	P (PVS1, PM2, PP5)	1
**F331**	CysKD	*PKD2*	# 613095 (AD)	Splicing (Ex. 10)	c.2020-2A>G p.? (het.)	0	P (PVS1, PM2, PP3, PP5)	1
**F363**	CysKD	*PKD2*	# 613095 (AD)	Stopgain (Ex. 2)	c.602G>A p.W201Ter (het.)	0.000001317	P (PVS1, PS4, PM2, PP5)	1

**F378**	CysKD	*PKD2*	# 613095 (AD)	Stopgain (Ex. 2)	c.602G>A p.W201Ter (het.)	0.000001317	P (PVS1, PS4, PM2, PP5)	1
**F1274**	CysKD	*PKD2*	# 613095 (AD)	Stopgain (Ex. 4)	c.916C>T p.R306Ter (het.)	0.000003718	P (PVS1, PS4, PM2, PP5)	1
**F634**	CysKD	*PKD2*	# 613095 (AD)	Stopgain (Ex. 6)	c.1480G>T p.E494Ter (het.)	0	P (PVS1, PM2, PP1, PP5)	2
**F631**	CysKD	*PKD2*	# 613095 (AD)	Stopgain (Ex. 6)	c.1480G>T p.E494Ter (het.)	0	P (PVS1, PM2, PP1, PP5)	2
**F354**	CysKD	*PKD2*	# 613095 (AD)	Stopgain (Ex. 12)	c.2286C>A p.Y762Ter (het.)	0.00000186	P (PVS1, PM2, PP5)	1
**F633**	CysKD	*PKD2*	# 613095 (AD)	Stopgain (Ex. 14)	c.2614C>T p.R872Ter (het.)	0.000006196	P (PVS1, PS3, PM2, PP5)	1
**F644**	CysKD	*PKD2*	# 613095 (AD)	Stopgain (Ex. 14)	c.2614C>T p.R872Ter (het.)	0.000006196	P (PVS1, PS3, PM2, PP5)	1
**F428**	CysKD	*PKD2*	# 613095 (AD)	In-frame deletion (Ex. 5)	c.982_984del p.328_328del (het.)	0.00000159	LP (PM1, PM2, PM4)	1
**F498**	CysKD	*PKD2*	# 613095 (AD)	Missense (Ex. 4)	c.964C>T p.R322W (het.)	0.000002479	P (PS3, PM2, PM5, PP3, PP5)	1
**F660**	CysKD	*PKD2*	# 613095 (AD)	Missense (Ex. 4)	c.964C>T p.R322W (het.)	0.000002479	P (PS3, PM2, PM5, PP3, PP5)	1
**F652**	CysKD	*PKD2*	# 613095 (AD)	Missense (Ex. 4)	c.974G>A p.R325Q (het.)	0.000001368	LP (PS3, PM2, PP3, PP5)	1
**F662**	CysKD	*IFT140*	# 614620 (AD)	Splicing (Ex. 10)	c.1010-1G>A p.? (het.)	0	LP (PVS1, PM2)	1
**F1242**	CysKD	*PKHD1*	# 606702 (AR)	Missense (Ex.50) Frameshift deletion (Ex. 5)	c.8068T>C:p.W2690R<br>c.338delG:p.G113Dfs*4 (Comp. het.)	0.00003098<br>0	LP (PM1, PM2, PM3, PM5, PP3, PP5)<br>LP (PVS1, PM2, PP5)	1
**F200**	CysKD	*ALG5*	# 604565 (AD)	Missense (Ex. 2)	c.235C>T<br>p.R79W (het.)	0	P (PS3, PP1-S, PM2, PP3)	4
**F350**	CysKD	*ALG5*	# 604565 (AD)	Missense (Ex. 2)	c.235C>T<br>p.R79W (het.)	0	P (PS3, PP1-S, PM2, PP3)	5
**F1269**	CysKD	*DNAJB11*	# 611341 (AD)	Stopgain (Ex. 2)	c.100C>T p.R34Ter (het.)	0.00000345	P (PVS1, PM2, PS4, PP5)	1
**F1271**	CysKD	*ALG9*	# 606941 (AD)	Missense (Ex. 10)	c.1102G>A p.E368K (het.)	0	LP (PM2, PP3_strong)	1
**F322**	NUC	*DYNC2H1*	# 603297 (AR)	Missense (Ex.85) Splicing (Ex. 65)	[c.12410C>G; p.P4137R]<br>[c.10042+2T>G 100% ESS] (Comp. het.)	0.00003101<br>0.00003418	LP (PM2, PM3, PP5) P (PVS1, PM2, PP3, PP5)	1
**Collagenopathies and Podocytopathies genes (Transcripts: *COL4A5* (NM_033380.3), *COL4A3* (NM_000091.5), *INF2* (NM_022489.4), *PAX2* (NM_000278.5), *RCAN1* (NM_004414.7))**								
**F100**	Alport/FSGS	*COL4A5*	# 303630 (XLD)	Missense (Ex. 31)	c.2605G>A p.G869R (het.)	0	P (PS4, PS3, PM1, PM2, PM5, PP2, PP3, PP5)	1
**F21**	Alport/FSGS	*COL4A5*	# 303630 (XLD)	Missense (Ex. 39)	c.3509G>A p.G170D (het.)	0	P (PM1, PM2, PP2, PP5_strong)	1
**F403**	Alport/FSGS	*COL4A5*	# 303630 (XLD)	In-frame deletion (Ex. 34)	c.2959_2976del p.987_992del (hemi.)	0	LP (PS1, PM2, PM4, PP5)	1
**F689**	Alport/FSGS	*COL4A5*	# 303630 (XLD)	Missense (Ex. 41)	c.3731G>A p.G1244D (hemi.)	0.000002199*	P (PS3, PM1, PM5, PP3_strong, PP5)	1
**F681**	Alport/FSGS	*COL4A3*	# 120070 (AD)	Splicing (Ex. 48)	c.4253-1G>A<br>p.? (het.)	0	P (PVS1, PM2, PP3, PP5)	1
**F859**	Alport/FSGS, NUC	*COL4A3*	# 120070 (AD)	Missense (Ex. 26)	c.1918G>A p.G640R (het.)	0.00001409*	LP (PS3, PM1, PP3, PP5)	2
**KF7**	Alport/FSGS	*COL4A3*	# 120070 (AD)	Missense (Ex. 31)	c.2452G>A p.G818R (het.)	0.00007353	LP (PS3, PM1, PP3_strong, PP5)	1
**KF4**	Alport/FSGS	*INF2*	# 610982 (AD)	Missense (Ex. 4)	c.653G>A p.R218Q (het.)	0	P (PS3, PM1, PM2, PM5, PP3, PP5)	1
**F899**	NUC	*INF2*	# 610982 (AD)	Missense (Ex. 6)	c.739G>C p.A247P (het.)	0	LP (PM2, PP3, PP1)	2
**F1227**	NUC	*INF2*	# 610982 (AD)	Missense (Ex. 4)	c.529C>T p.R177C (het.)	0	LP (PM1, PM2, PM5, PP3, PP5)	1
**F307**	GN	*PAX2*	# 167409 (AD)	Missense (Ex. 2)	c.70G>C p.G24R (het.)	0	LP (PM1, PM2, PM5, PP2, PM5, PP3)	1
**F111**	Alport/FSGS	*RCAN1*	# 602917 (AD)	Missense (Ex. 3)	c.485T>C p.I162T (het.)	0.000007	P (PS3, PP1_Strong, PP3)	6
**Tubulointerstitial Disease Genes (Transcripts: *MUC1* (NM_001204286.1), *UMOD* (NM_003361.4), *HNF1B* (NM_000458.4))**								
**F860**	TIKD	*MUC1*	# 158340 (AD)	Frameshift insertion	c. ins(3n+1) in VNTR<br>p. MUC1fs (het.)	0	P (PVS1, PS1, PS3, PP1)	1
**F386**	TIKD	*MUC1*	# 158340 (AD)	Frameshift insertion	c. ins(3n+1) in VNTR<br>p. MUC1fs (het.)	0	P (PVS1, PS1, PS3, PP1)	1
**F309**	NUC	*MUC1*	# 158340 (AD)	/	Kidney Biopsy MUC1fs protein	/	Pathogenic	1
**F165**	TIKD	*UMOD*	# 191845 (AD)	Missense (Ex. 3)	c.821A>G p.Y274C (het.)	0.000001861	LP (PM1, PP2, PP3_strong)	1
**F922**	TIKD	*UMOD*	# 191845 (AD)	Missense (Ex. 3)	c.184A>C p.T62P (het.)	0.0005459	P (PS3, PS4, PP3, PP5)	2
**F952**	NUC	*UMOD*	# 191845 (AD)	Missense (Ex. 3)	c.184A>C p.T62P (het.)	0.0005459	P (PS3, PS4, PP3, PP5)	2
**F954**	TIKD	*UMOD*	# 191845 (AD)	Missense (Ex. 3)	c.184A>C p.T62P (het.)	0.0005459	P (PS3, PS4, PP3, PP5)	1
**F985**	TIKD	*UMOD*	# 191845 (AD)	Missense (Ex. 3)	c.184A>C p.T62P (het.)	0.0005459	P (PS3, PS4, PP3, PP5)	1
**F1015**	TIKD	*UMOD*	# 191845 (AD)	Missense (Ex. 3)	c.184A>C p.T62P (het.)	0.0005459	P (PS3, PS4, PP3, PP5)	1

**F1077**	TIKD	*UMOD*	# 191845 (AD)	Missense (Ex. 3)	c.184A>C p.T62P (het.)	0.0005459	P (PS3, PS4, PP3, PP5)	1
**F622**	TIKD	*UMOD*	# 191845 (AD)	Missense (Ex. 3)	c.184A>C p.T62P (het.)	0.0005459	P (PS3, PS4, PP3, PP5)	1
**F90**	TIKD	*HNF1B*	# 189907 (AD)	Splicing (Ex. 2)	c.544+3_544+6del 75% ESS (het.)	0	P (PVS1, PM2, PP5)	1
**F306**	NUC, CAKUT	*HNF1B*	# 189907 (AD)	Frameshift deletion (Ex. 6)	c.1333_1334del p.Ala445fs (het.)	0	P (PVS1, PM2, PP1_supp)	3
**NCNL / Tubulopathy Genes (Transcripts: *CLCN5* (NM_001127898.4), *SLC26A1* (NM_022042.4), *SLC4A1* (NM_000342.4))**								
**F182**	NUC	*CLCN5*	# 300008 (XLR)	Frameshift deletion (Ex. 13)	c.1938del p.F646LfsTer10	0	LP (PVS1, PM2)	1
**F935**	Others	*CLCN5*	# 300008 (XLR)	Splicing (Ex. 6)	c.166-2A>T p.?	0	P (PVS1, PM2, PP3)	1
**F1239**	NUC	*SLC26A1*	# 610130 (AR)	Missense (Ex. 3 and 2)	c.1073C>T; p.S358L c.554C>T; p.T185M	0.0003832 0.0002944	P (PS3, PP3_mod, PP5, PM3) and P (PS3, PP3_mod, PP5, PM3)	1
**F946**	Others	*SLC4A1*	# 109270 (AD)	Missense (Ex. 14)	c.1765C>T p.R589C	0	LP (PM1, PM2, PM5, PP3, PP5)	1

Abbreviations: ACMG: American College of Medical Genetics and Genomics; AD/AR: autosomal dominant/recessive inheritance mode; AF: Allelic frequency; CAKUT: Congenital anomalies of the kidneys and urinary tracts; CNV: copy number variant; Comp. het.: Compound heterozygote; CysKD: cystic kidney disease; Ex.: exon; hemi.: hemizygous; het.: heterozygote; ID: identifier; FSGS: focal segmental glomerulosclerosis; gnomAD: The Genome Aggregation Database version 4; GN; glomerulonephritis; LP: likely pathogenic; NUC: Nephropathy of undetermined cause; OMIM: Online Mendelian Inheritance in Man; P: pathogenic; PKD: cystic kidney disease; TIKD: tubulointerstitial kidney disease; VNTR: Variable number of tandem repeats; XLD/XLR: X-linked dominant and recessive inheritance mode

* This variant is covered in fewer than 50% of individuals in gnomAD v4.0.0 exomes. Allele frequency estimates may not be reliable.

Table S2 | Genetic Variants Distribution in Patients Over 60 Years. The age groups of 217 sequenced individuals were categorized based on the gene group grouping, regardless of whether the causative variants had been identified or if there was no molecular diagnosis (NMD). ADTKD: autosomal dominant tubulointerstitial kidney disease; n: number; NCNL: nephrocalcinosis and nephrolithiasis.

**Distribution of Gene Groups by Age**						
**Age Groups**, years	**Cystic Kidney / Ciliopathies Genes** (n=133)	**ADTKD Genes** (n=17)	**Collagenopathies / Podocytopathies Genes** (n=18)	**NCNL / Tubulopathy Genes** (n=4)	**NMD** (n=93)	**Total**
60 - <65	18 (13.53)	1 (5.88)	0 (0)	1 (25)	7 (7.53)	27 (10.19)
65 - <70	41 (30.83)	4 (23.53)	6 (33.33)	2 (50)	26 (27.96)	79 (29.81)
70 - <75	36 (27.07)	4 (23.53)	6 (33.33)	0 (0)	26 (27.96)	72 (27.17)
75 - <80	19 (14.29)	6 (35.29)	4 (22.22)	0 (0)	23 (24.73)	52 (19.62)
80 and over	19 (14.29)	2 (11.76)	2 (11.11)	1 (25)	11 (11.83)	35 (13.21)

Table S3 | Diagnostic Yield based on Age Groups and Age at Disease Onset

**Age Groups**	**% of Diagnostic Yield**	**P value [a]**	**Age at Disease Onset**	**% of Diagnostic Yield**	**P value [a]**
**≥80 Years (n=35) vs. <80 Years (n=230)**	68.6% vs. 63.9%	0.706	≤ 30 Years (n=198) vs. > 30 Years (n=67)	66.2% vs. 59.7%	0.376
**≥75 Years (n=87) vs. <75 Years (n=178)**	60.9% vs. 66.3%	0.414	≤ 40 Years (n=157) vs. > 40 Years (n=108)	67.5% vs. 60.2%	0.241
**≥70 Years (n=159) vs. <70 Years (n=106)**	61.6% vs. 68.8%	0.241	≤ 50 Years (n=100) vs. > 50 Years (n=165)	64% vs. 64.8%	0.895
**≥65 Years (n=238) vs. <65 Years (n=27)**	63.5% vs. 74.1%	0.299	≤ 60 Years (n=214) vs. > 60 Years (n=51)	66.8% vs. 54.9%	0.142

[a] Fisher Exact Test

Table S4 | Patients with Modified Diagnosis

Family ID / Individual ID	Gene / #OMIM Associated Condition	A priori diagnosis: Clinical findings	Rational [a]
**F307 / 10285**	*PAX2* #120330 (AD) Papillorenal syndrome	71 M, presented at age 42 years nephrotic range proteinuria, with failed attempt of kidney biopsy. Progressed to KF at age 46 years with impression of chronic glomerulonephritis and hypertension as a prior diagnosis	The diagnosis of papillorenal syndrome facilitated the establishment of a precise diagnosis and enabled the reversal of phenotyping, eye examination, and familial cascade testing
**F306 / 28192**	*HNF1B* #137920 Renal cysts and diabetes syndrome	77 F, presented non-proteinuric CKD at age 50 years. Small kidneys not allowing kidney biopsy, keeping with non-specific diagnosis of nephropathy of undetermined cause	Renal features could be explained precisely as the diagnosis has been established, allowing investigation for extrarenal association and follow-up after kidney transplantation
**F100 / 10530**	*COL4A5* #301050 (XLD) Alport syndrome 1	77 F, presented proteinuric CKD at age 30 years, haematuria, and hypertension. Progressed to KF at age 39 years.	Identifying the *COL4A5* missense variant allowed the establishment of renal features of Alport syndrome.
**F860 / 11062**	*MUC1* #174000 (AD) ADTKD	70 F, referred for slowly progressive non-proteinuric CKD stage 3a at the age of 65 years, and a strong family history of kidney disease. Her father died young, secondary to KF, and her daughter has progressive CKD in her 30s with a kidney biopsy revealing tubulointerstitial changes	The identification of the *MUC1* pathogenic variant confirms the diagnosis of ADTKD-*MUC1*, which represents a specific subcategory within a larger group of clinically suspected diseases and facilitate the understating of disease variability within family
**KF4 / 22516**	*INF2* #613237 (AD) Glomerulosclerosis, focal segmental, 5	85 F progressed to KF at age 77 years with the impression of hereditary nephritis and a strong family history of kidney disease.	The identification of the pathogenic variant in the *INF2* gene led to a precise diagnosis and subsequently reclassified the initial diagnosis.
**F309 / 10590**	*MUC1* #174000 (AD) ADTKD	73 M, diagnosed with hypertension, gout, and nephropathy of undetermined cause at 53 years. Kidney biopsy revealed non-specific vascular and interstitial changes. His kidney function declined, reaching KF at the age of 67 years with the impression of unknown cause of KF	Despite the lack of insight provided by the MPS data analysis and *MUC1* genotyping, identifying the mutant MUC1 protein through immunostaining of the MUC1fs protein helped establish the diagnosis and facilitate decisions pertaining to kidney transplantation.
**F322 / 10011**	*DYNC2H1* #613091 (AR) Short-rib thoracic dysplasia 3 with or without polydactyly	75 F, referred due to progressive CKD and the presence of small-sized kidneys with scattered cysts. In addition, she was diagnosed with retinitis pigmentosa, dextrocardia, and liver dysfunction. The cause of CKD remained undetermined when she reached KF in her seventies.	Identified the molecular diagnosis for the cause that remained unknown at the time of disease progression.
**KF7 / 22517**	*COL4A3* #104200 (AD) Alport syndrome 3A	73 F, diagnosed with haematuric CKD at age 12 years. She underwent unilateral nephrectomy secondary to kidney tuberculosis, for which her kidney function gradually declined until she reach KF at 61 years with the impression of adaptive FSGS.	The detection of the monoallelic *COL4A3* variant provided further insight into the underlying cause of disease progression, which had been

			hypothesized before genetic testing. This identification refined the clinical diagnosis.
**F403 / 10274**	*COL4A5* #301050 (XLD) Alport syndrome 1	At the age of 68 M, was identified as having advanced CKD and hypertension. Before this, he had been diagnosed with sensorineural hearing loss and gout in his twenties. Secondary FSGS and chronic interstitial changes were identified in native kidney biopsy. He was diagnosed with "hereditary nephritis" or "hereditary TKD" by the time he developed KF at the age of 57.	Identifying the pathogenic variant in the *COL4A5* gene resulted in an accurate diagnosis and subsequently allowed for identifying a specific cause among a wide range of potential clinical scenarios.
**F182 / 10602**	*CLCN5* #300009 (XLR) Dent disease 1	68 M presented with KF requiring kidney replacement therapy	Identified the molecular diagnosis for the cause that remained unknown at the time of disease progression.
**F946 / 10839**	*SLC4A1* #179800 (AD) Distal renal tubular acidosis 1	A 66-year-old individual has been diagnosed with recurring nephrolithiasis and urinary infections. She experienced a gradual decline in kidney function, leading to KF at the age of 58. The primary cause of CKD was determined to be nephrolithiasis.	Identifying the molecular diagnosis allowed for identifying a specific cause among a wide range of potential clinical scenarios.
**F899 / 10808**	*INF2* #613237 (AD) Glomerulosclerosis, focal segmental, 5	A 67-year-old individual was diagnosed with proteinuria during pregnancy at the age of 22 and lost of follow-up until presenting at age 43 with hypertension emergency and advanced CKD. The native kidney biopsy showed significant vascular changes and chronic interstitial abnormalities. The primary cause leading to CKD was thought to be hypertensive nephrosclerosis upon the commencement of dialysis at the age of 43.	Identified the molecular diagnosis for the cause that remained unclear at the time of disease progression.
**F899 / 87082**	*INF2* #613237 (AD) Glomerulosclerosis, focal segmental, 5	Presented at age 38 years with advanced CKD, nephrotic-range proteinuria and hypertension emergency. His relative (10808) has been advised to undergo a renal evaluation at that time.	Identifying the molecular diagnosis allowed for identifying a specific cause among a wide range of potential clinical scenarios.
**F1227 / WES139**	*INF2* #613237 (AD) Glomerulosclerosis, focal segmental, 5	67 F, presented with hypertension and proteinuric CKD. Kidney biopsy revealed vascular changes and secondary FSGS. Family history was unknown. At the age of 55, she experienced a decline in kidney function and had a pre-emptive kidney transplant due to a primary diagnosis of hypertensive nephrosclerosis.	Identified the molecular diagnosis for the cause that remained unclear at the time of disease progression.
**F922 / 11253**	*UMOD* (p.T62P) #162000 (AD) ADTKD	70 M, presented with hypertension and atrial fibrillation at the age of 54 years. eGFR was reduced at 29 ml/min and mild proteinuria. No prior kidney biopsy. The cause of CKD remained undetermined when he reached KF at the age of 65 years.	Identified the molecular diagnosis for the cause that remained unclear at the time of disease progression. Renal features associated with intermediate *UMOD* allelic effect.
**F1242 / WES98**	*PKHD1* #263200 (AR) Polycystic kidney disease 4, with	62 F, referred for cystic kidney disease with no family history. eGFR was reduced at 34 ml/min.	Cystic kidney disease is a characteristic of autosomal recessive polycystic kidney disease. The molecular diagnosis helps identify a specific cause from various clinical scenarios of cystic kidneys

	or without hepatic disease		and allows for further evaluation of phenotypic involvements.
**F622 / 10633**	*UMOD* (p.T62P) #162000 (AD) ADTKD	76 M, presented with CKD and multiple kidney cysts that were not suitable for kidney biopsy. The cause of the cysts remains unknown, but it is presumed to be IgA nephropathy. His eGFR gradually decreased, leading to KF when he was 68.	Identified the molecular diagnosis for the cause that remained unclear at the time of disease progression. Renal features associated with intermediate *UMOD* allelic effect.
**F952 / 11197**	*UMOD* (p.T62P) #162000 (AD) ADTKD	76 M, presented with hypertension, gout, and CKD at age 54 years. Kidney biopsy revealed arteriosclerosis and secondary FSGS which were presumed to be the cause of CKD at the time of KF at age 60 years.	Identified the molecular diagnosis for the cause that remained unclear at the time of disease progression. Renal features associated with intermediate *UMOD* allelic effect.
**F952 / WES111**	*UMOD* (p.T62P) #162000 (AD) ADTKD	A 76-year-old male patient presented with hypertension, gout, and CKD at the age of 54. The kidney biopsy showed arteriosclerosis and secondary FSGS, which were assumed to be the underlying cause of CKD leading to KF at the age of 60 years.	Identified the molecular diagnosis for the cause that remained unclear at the time of disease progression. Renal features associated with intermediate *UMOD* allelic effect.
**F1239 / WES94**	*SLC26A1* #167030 Nephrolithiasis, calcium oxalate, 1	a 63-year-old female presented with recurrent non-obstructive nephrolithiasis, urinary infections, and proteinuria. Sonography revealed normal-sized kidneys. In addition, she has reported bilateral sensorineural deafness since childhood, indicating hearing aids at age 50. No further evidence of systemic involvement was identified. At the last follow-up, she exhibited renal dysfunction at 63 years old (eGFR of 45 mL/min/1.73 m^2).	The molecular diagnosis enabled the identification and partial explanation of the cause of CKD.
**F859 / 11523**	*COL4A3* #104200 (AD) Alport syndrome 3A	76 F, referred for CKD evaluation, assumed secondary to hypertension and non-steroidal anti-inflammatory meds. The eGFR was 45 ml/min at the age of 73. The urine dip test revealed moderate red blood cells (++) and a small amount of protein (+). A kidney biopsy has not been performed previously.	The molecular diagnosis for the assumed cause of disease progression initially attributed to hypertension, has been identified.

[a] The rationale for modifying the clinical diagnosis in light of genetic findings relied on whether genetic testing yielded novel insights that differed from the *a priori* diagnosis regarding the underlying disease mechanism. **Abbreviations:** ADTKD: autosomal dominant tubulointerstitial kidney disease; CKD: chronic kidney disease; F: female; FSGS: focal segmental glomerulosclerosis; ID: identifier; KF: kidney failure; M: male; MPS: massive parallel sequencing; OMIM: Online Mendelian Inheritance in Man

Table S5 | Kidney Survival Predictors in Older Adults with Suspected Genetic Kidney Disease. Univariate and multivariate Cox Proportional Hazard models were employed to investigate the association between predictor variables and time to kidney failure (kidney survival). The assumption of proportional hazard was also tested and confirmed.

**Variables**	**Univariate analysis**		**Multivariate analysis**	
	**HR (95% CI)**	***P* Value**	**HR (95% CI)**	***P* Value**
**Sex (Male)**	1.38 (1.03 - 1.83)	**0.026**	1.75 (1.23 - 2.52)	**0.002**
**Onset of Disease (<60 Years)**	3.64 (2.41 - 5.52)	**<0.001**	3.14 (1.7 - 5.79)	**<0.001**
**Genetic Testing (Positive Dx)**	1.29 (0.95 - 1.74)	0.097	/	/
**Family history of CKD (Yes)**	1.06 (0.72 - 1.55)	0.761	/	/
**Type of Variants (Variants causing "later-onset" phenotypes) [a]**	0.36 (0.2 - 0.62)	**<0.001**	0.52 (0.27 - 0.98)	**0.043**
***A priori* diagnosis [b]**				
**Alport/FSGS (vs. CysKD)**	0.74 (0.43 - 1.27)	0.281	0.85 (0.44 - 1.68)	0.655
**TIKD (vs. CysKD)**	0.46 (0.21 - 0.98)	**0.046**	0.82 (0.34 - 1.94)	0.655
**GN (vs. CysKD)**	1.66 (0.98 - 2.79)	0.057	4.56 (1.03 - 20.18)	**0.046**
**NUC (vs. CysKD)**	1.19 (0.76 - 1.86)	0.437	1.73 (0.89 - 3.34)	0.101
**Others (vs. CysKD)**	0.91 (0.46 - 1.80)	0.801	1.36 (0.42 - 4.38)	0.598

[a] Variants causing "later-onset" phenotypes include monoallelic *COL4A3* and *COL4A5* variants in female patients, ADPKD-like disease spectrum, and *UMOD* p.Thr62Pro variant, were compared to genetic variations typically associated with genetic kidney disease, such as variants in the *PKD1*, *PKD2*, *INF2, MUC1*, canonical *UMOD* variants, and *HNF1B* genes among others.

[b] Evaluation of disease progression was conducted by comparing the primary clinical presentation categories (*a priori* diagnosis) to cystic kidney disease as a reference, as the most prevalent clinical presentation.

CI: Confidence Interval; CysKD: cystic kidney disease; Dx: Diagnosis; FSGS: focal segmental glomerulosclerosis; HR: Hazard Ratio; GN; glomerulonephritis; NUC: Nephropathy of undetermined cause; TIKD: tubulointerstitial kidney disease

Table S6 | Phenotypes of *UMOD* p.T62P carriers

Family ID (Individual ID)	Sex (Age at last FU, years)	Age of Onset (Years)	the "*a priori*" diagnosis category	Progression to Kidney Failure (if yes; Age at Kidney Failure (years)	eGFR (Age at measurement (years))	Kidney biopsy results, if performed	Other conditions (Age)	Genetic Testing Platform
**F952 (11197)**	M (76)	56	NUC	KF (60)		Yes: arteriosclerosis and secondary FSGS	Gout (49), Hypertension (54), Bronchiectasis (56)	WES
**F952 (WES111)**	F (83)	45	NUC	KF (65)		No	Hypertension (55), Atrial fibrillation (68), Gout (70), Diabetes Mellites (67)	WES
**F985 (WES80)**	M (63)	59	TIKD	CKD	13 (63)	Yes: tubulointerstitial changes of uncertain cause	Hypertension (60)	TGP
**F622 (10633)**	M (76)	67	TIKD	KF (68)		No	Gout (55), , TIA (56), Atrial fibrillation (60), Hypertension (62)	TGP
**F954 (11215)**	M (78)	68	TIKD	KF (74)		Yes: tubulointerstitial changes	Rheumatic fever (16), Gout (30), Ebstein's anomaly (60), Hypertension (68)	WES
**F1015 (11463)**	M (69)	67	TIKD	KF (69)		Yes: extensive tubulointerstitial changes	Hypertension (61), Atrial fibrillation (62), COPD (70), osteoporosis (71)	WES
**F922 (11253)**	M (70)	60	TIKD	KF (65)		Yes: tubulointerstitial changes	Hypertension (54), Atrial fibrillation (62), occipital stroke (65)	WES
**F922 (WES30)**	F (68)	62	TIKD	KF (63)		No	Hypertension (45), Migraine (55)	WES
**F1077 (WES62)**	F (76)	70	TIKD	CKD	26 (75)	No	hypertension (60), hyperthyroidism (66), Diabetes mellitus (75)	WES

Abbreviations: CKD: chronic kidney disease; COPD: chronic obstructive pulmonary disease; F: female; FU: follow-up; ID: identifier; KF: kidney failure; M: male; NUC: Nephropathy of undetermined cause; eGFR: estimated glomerular filtration rate as measure by the EPI equation; FSGS: focal segmental glomerulosclerosis; TIA: transient ischemic attack; TIKD: tubulointerstitial kidney disease TGP: targeted gene panel (as per PMID: 33454723); WES: whole exome sequencing;
